# Supplementary material for: A single-cell atlas of pig gastrulation as a resource for comparative embryology
Source: Nat Commun. 2024 Jun 18;15:5210. doi: 10.1038/s41467-024-49407-6 (PMC11189408; doi:10.1038/s41467-024-49407-6)
Supplement: Supplementary file 1 — Supplementary Information [file 41467_2024_49407_MOESM1_ESM.pdf]

REAGENT or RESOURCE	Antibody dilution used	SOURCE	IDENTIFIER
Primary Antibodies			
TBXT (Goat)	1:500	R&D	Cat# AF2085, RRID:AB_2200235
SOX17 (Rabbit)	1:500	Abcam	Cat# ab224637, RRID:AB_2801385
SOX17 (Goat)	1:750	R&D systems	Catalog #: AF1924, RRID:AB_355060
FOXA2 (Mouse)	1:750	Abnova	Cat# H00003170-M10, RRID:AB_534871
SOX2 (Rat)	1:5000	eBioscience	Cat# 14-9811-80, RRID:AB_11219070
SNAI1 (Rabbit)	1:200	Cell Signaling	Cat# 3879, RRID:AB_2255011
NANOG (Rabbit)	1:500	PeproTech	Cat# 500-P236, RRID:AB_2929968
Secondary Antibodies			
Alexa Fluor™ 488 (Donky Anti-Mouse)	1:500	Invitrogen	Cat# A-21206
Alexa Fluor™ 555 (Donky Anti-Rabbit)	1:500	Invitrogen	Cat# A-31572, RRID AB_162543
Alexa Fluor™ 555 (Donky Anti-Rat)	1:500	Invitrogen	Cat# A78945, RRID:AB_2910652
Alexa Fluor™ 647 (Donky Anti-Goat)	1:500	Invitrogen	Cat# A-21447, RID AB_2535864
IHC reagents			
PBS tablets		OXOID	Cat# BR0014G
Triton-X		Sigma-Aldrich	Cat# 9036-19-5
Flourosheild with Dapi		Sigma-Aldrich	Cat# F657
Cell lines			
EDSCL4 (Porcine)		Kinoshita et al 2021	
EDSCL7 (Porcine)		Kinoshita et al 2021	
HNES1 (human)		Guo et al 2016	
H9 (human)		WiCell	
Cell culture reagents and consumables			
DMEM/F12 medium		Gibco	Cat# 21103049
Neurobasal medium		Gibco	Cat# 11320074
B-27 supplement		Gibco	Cat# 17504001
N-2 supplement		Gibco	Cat# 17502001
GlutaMAX		Gibco	Cat# 35050038
2-Mercaptoethanol (50 mM)		Gibco	Cat# 31350010
Recombinant human Activin A protein		Qkine	Cat# Qk001
Recombinant zebrafish FGF2 protein		Qkine	Cat# Qk002
XAV939 (WNT inhibitor)		Sigma-Aldrich	Cat# X3004
Y-27632 dihydrochloride (ROCKi)		Torcris	Cat# 1254
L511-E8-silk (iMatrix-511)		AMSBIO	Cat# AMS.892 021
TRYPLE EXPRESS		Gibco	Cat# 12604013
CTS™ Synth-a-Freeze™ Medium		Gibco	Cat# A1371301
NutriFreez D10		Sartorius	Cat# 05-713-1E
96-Well CytoOne Plate, TC-Treated		Starlabs	Cat# CC7682-7596
Bovine Serum Albumin solution		Sigma-Aldrich	Cat# A8412

Supplementary Table 1: Summary of reagents used within this study

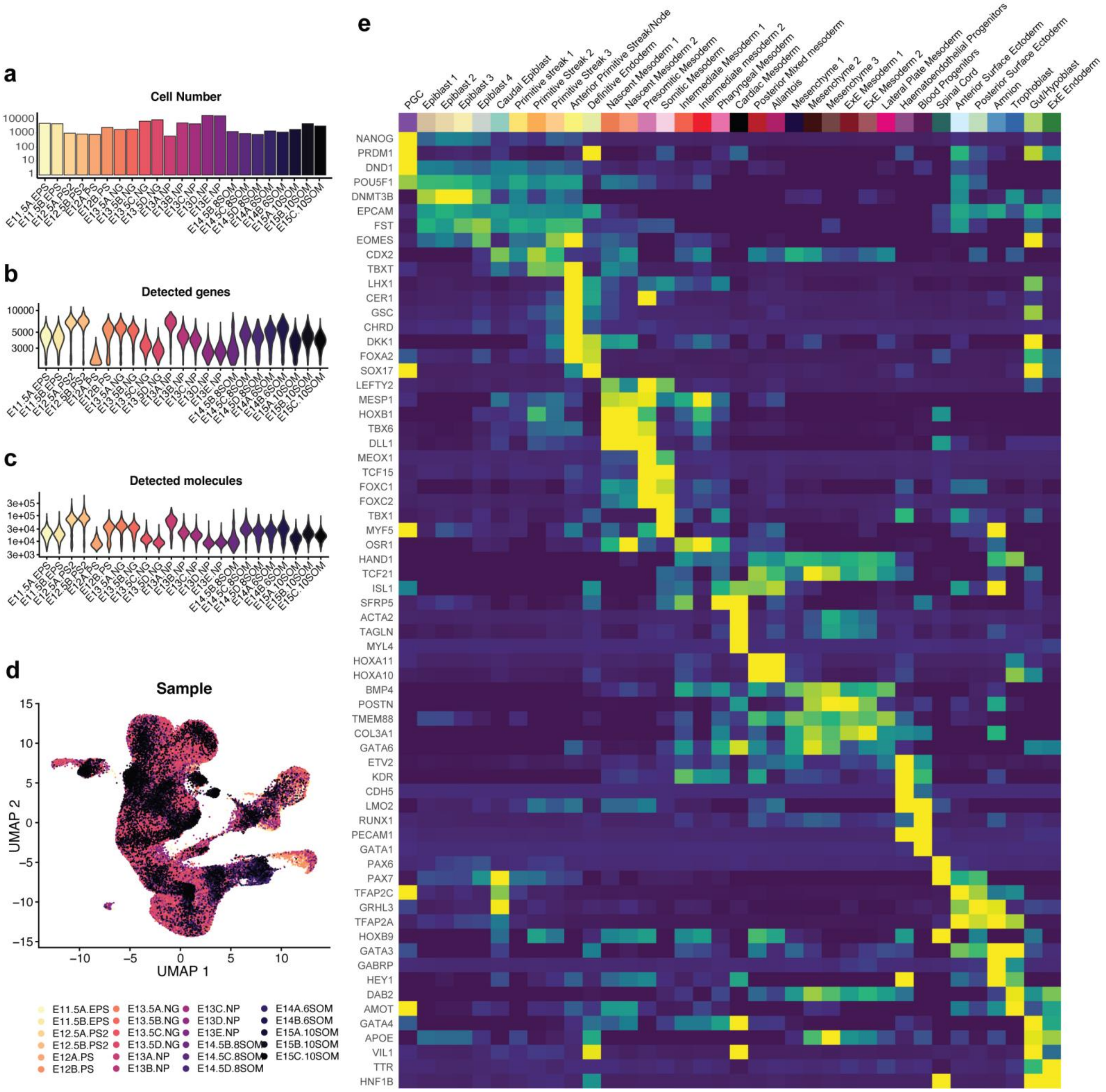

Supplementary Fig. 1. Cell distribution per sample and gene markers used to define cell identity. a, Number of cells captured in this study in each sample used in this atlas. E, Embryonic day. **b**, Violin plots illustrating the number of detected genes per cell per sample. **c**, The number of molecules detected per cell per sample. **d**, UMAP plot showing atlas cells from Fig.1c) coloured by sample, showing consistency across replicates from the same stage. **e**, Heatmap showing the markers used for cell type identification, a full list of these markers and the source publications are available in Supplementary Table 1. ExE; Extra-embryonic, PGC; Primordial germ cell.

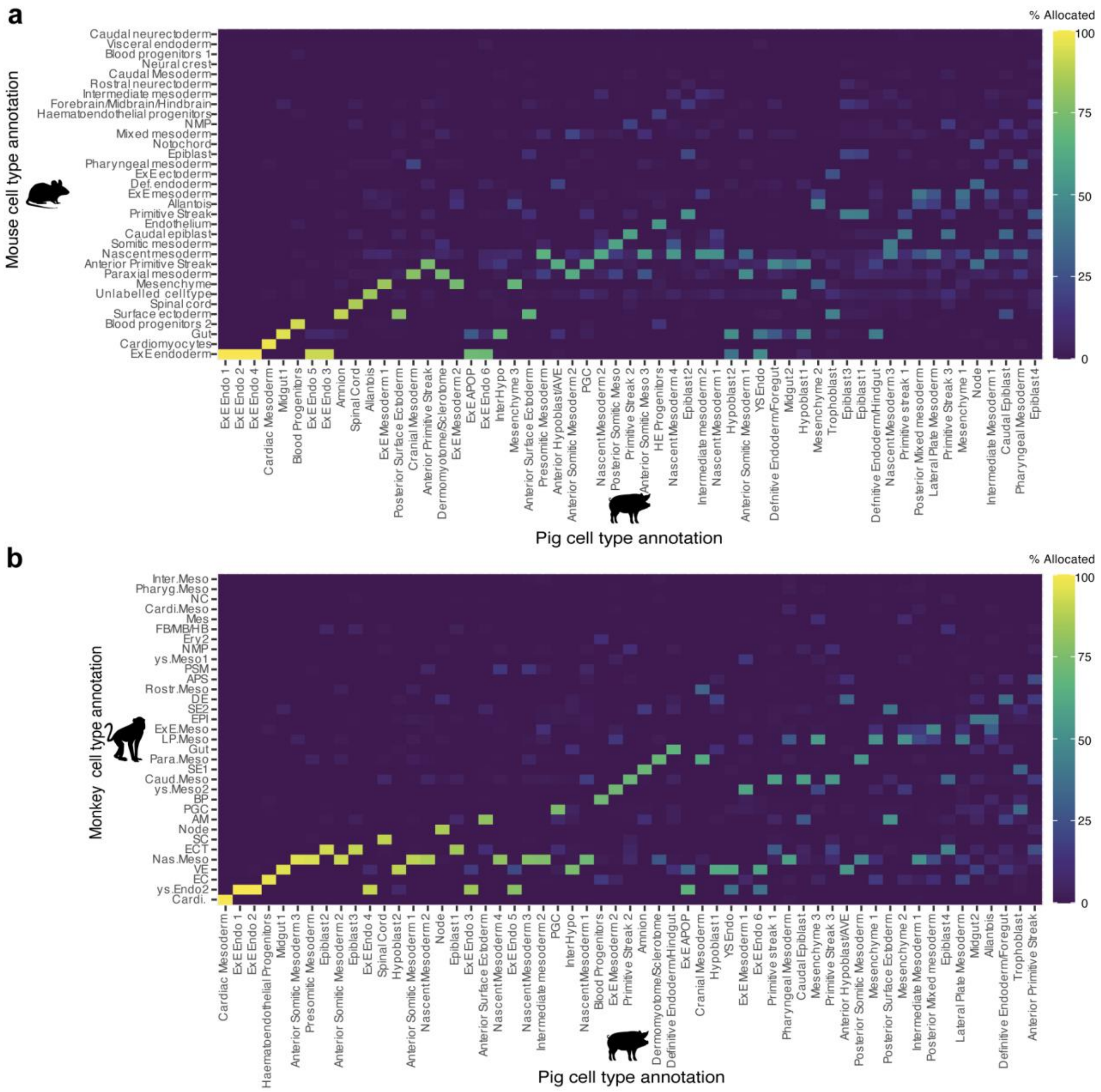

Supplementary Fig. 2. Comparisons of mouse and monkey cell annotations. a, Heat map showing the percentage of pig cells of a given cell type allocated as a particular mouse cell type after label transfer. **b,** Heat map showing the percentage of a given pig cell type allocated as a particular monkey cell type by label transfer. A percentage of 100 indicates that all of the cells of a given cell type were predicted to be analogous to the cell identity in the queried organism.

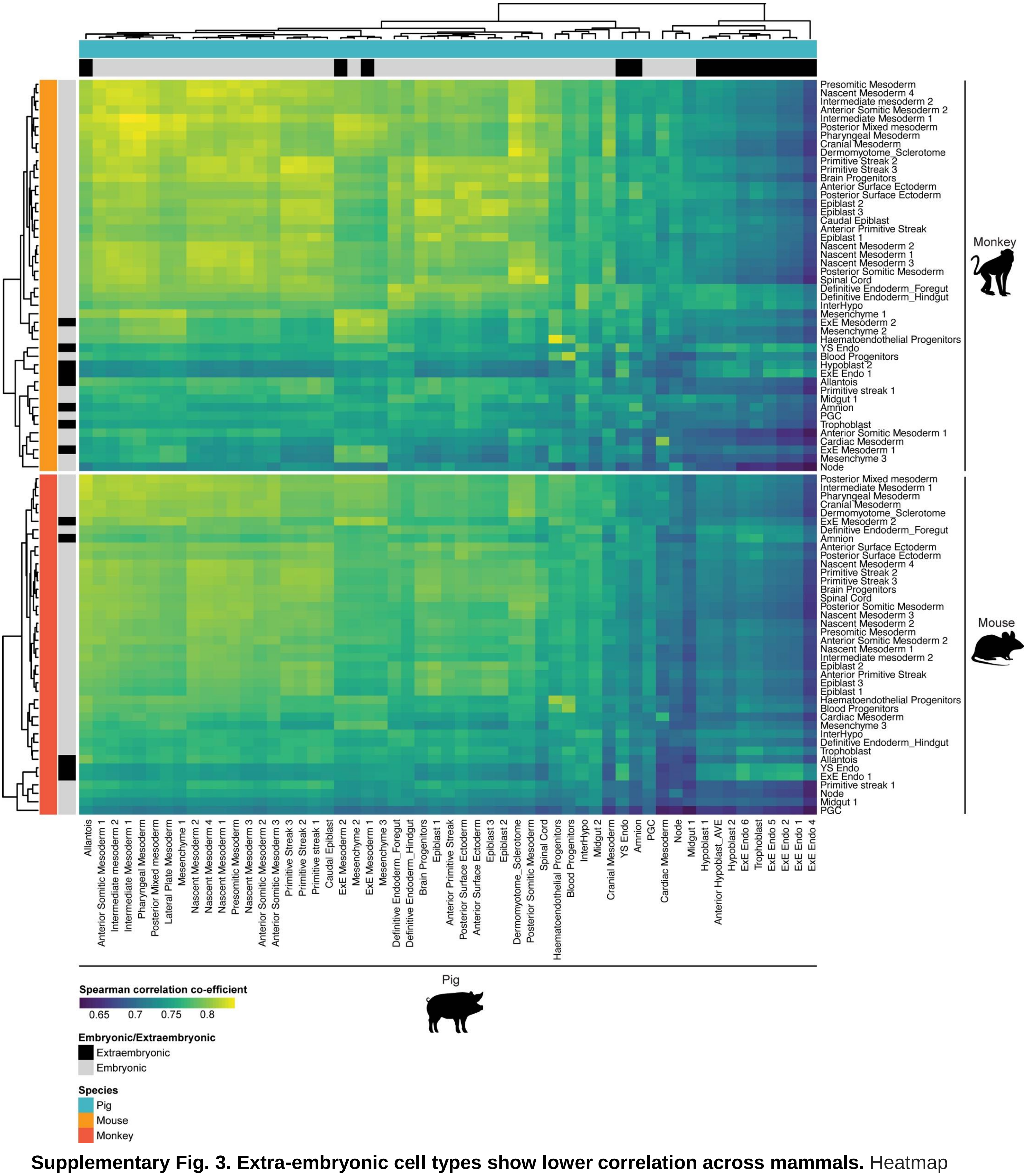

Supplementary Fig. 3. Extra-embryonic cell types show lower correlation across mammals. Heatmap showing Spearman correlation coefficient between the transcriptomes of matched cell types in pig, monkey and mouse embryos.

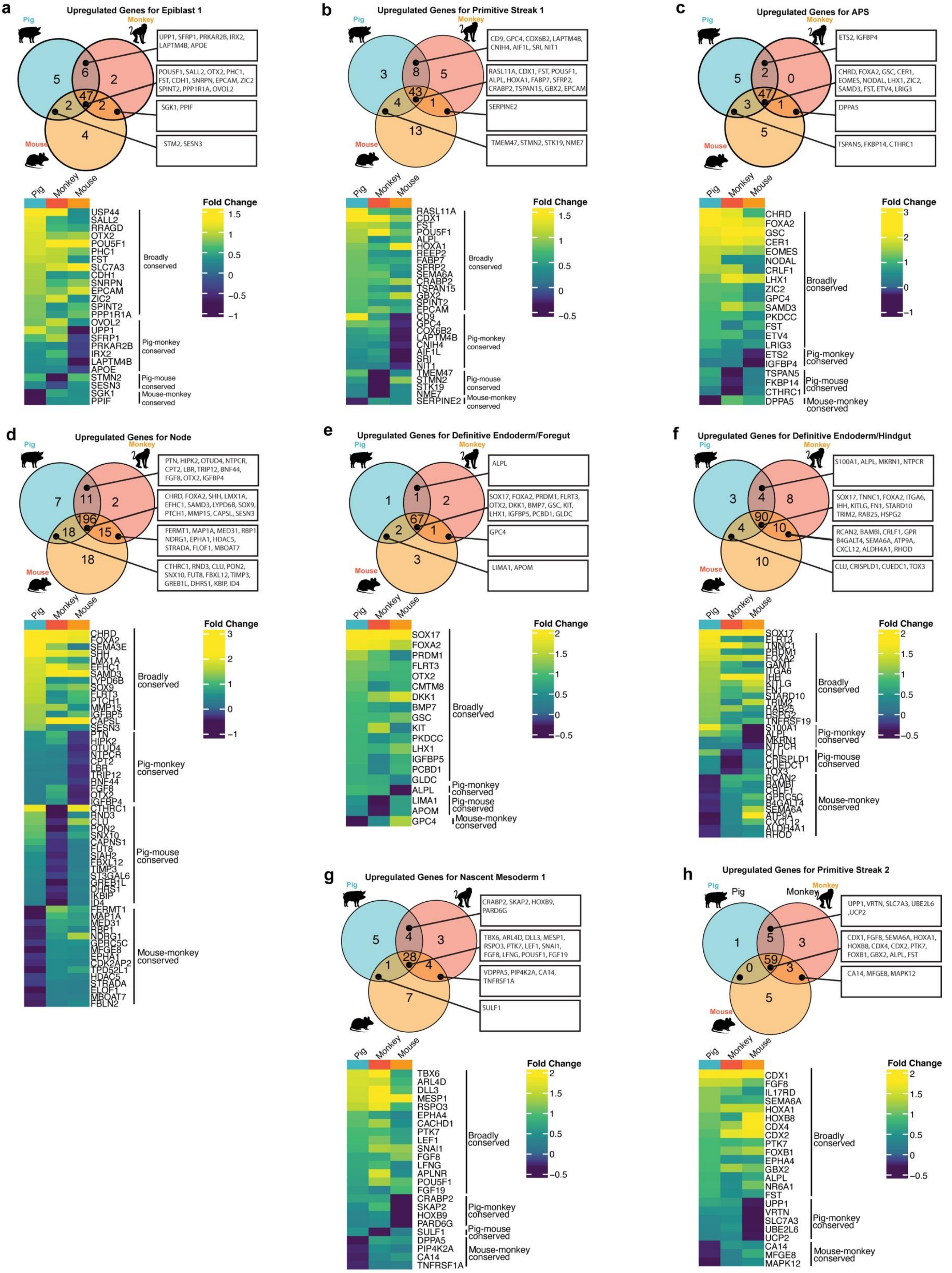

Supplementary Fig. 4. a, Cell type-specific gene expression programs are highly conserved. a-h, Venn diagrams and heatmaps showing unique and overlapping cell type-specific genes in pig, monkey and mouse. The heatmaps illustrate the average fold change in gene expression within the cell type of interest, compared to the mean fold change across the remaining cell types for a specific species. APS, Anterior primitive streak.

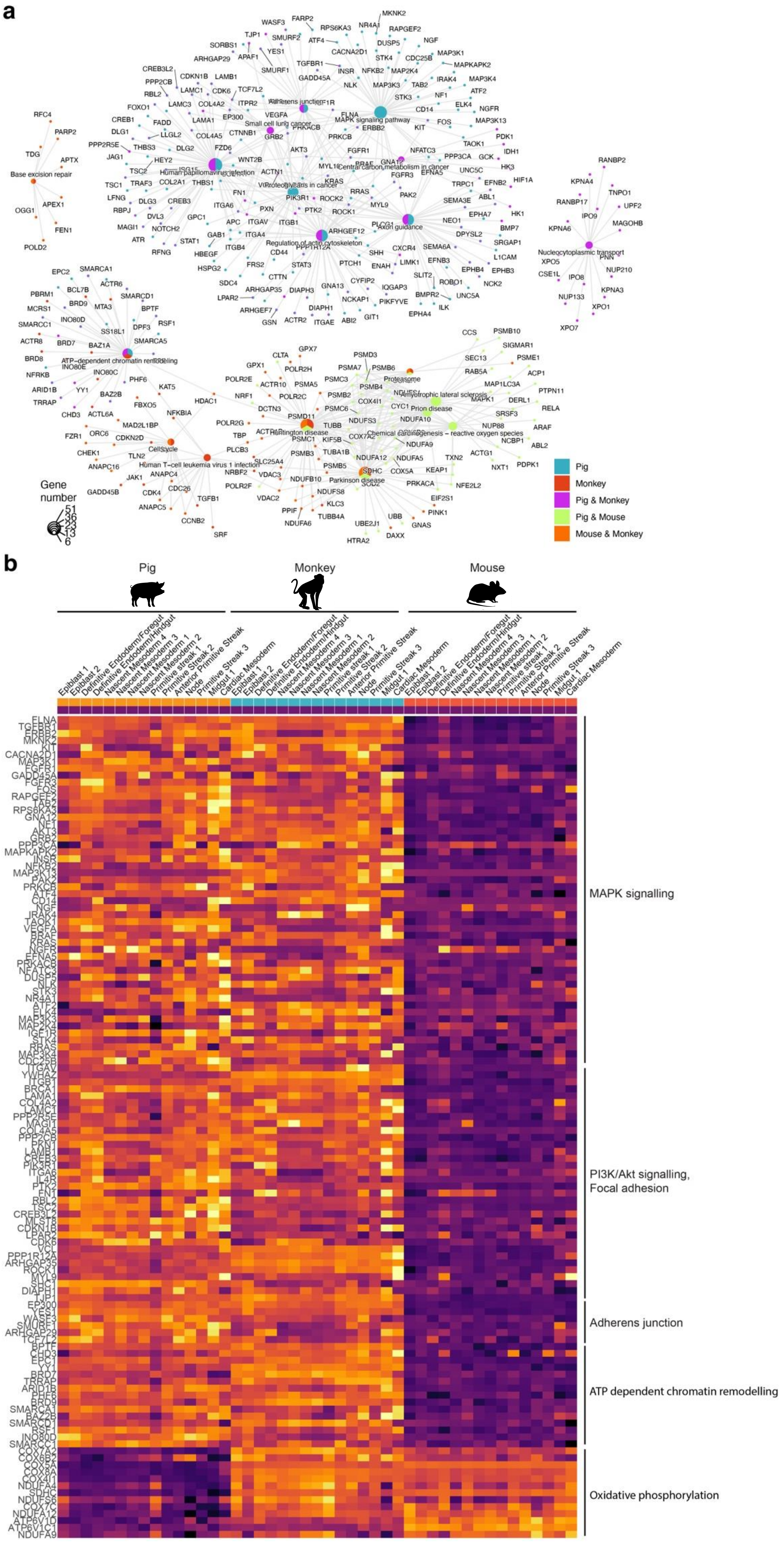

Supplementary Fig. 5. Broad differences in early cell behavior are observable across mammals. a, Plot of Cnet illustrating the enriched KEGG terms and associated genes found in both unique and shared expression profiles among pig, monkey, and mouse epiblast cells. **b,** Heat map of selected KEGG terms-associated gene expression in early pig, monkey and mouse cell types.

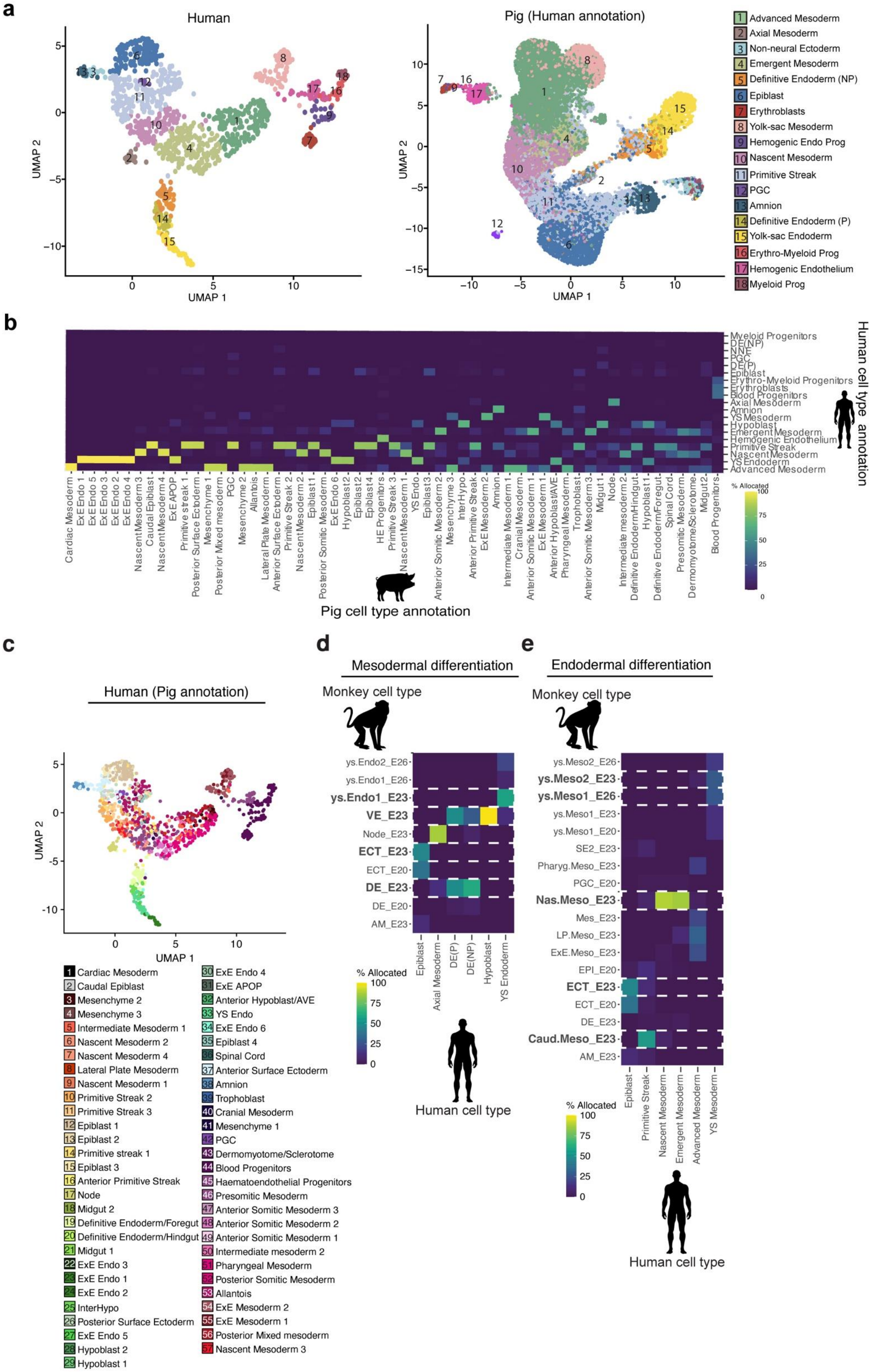
a
Human
Pig (Human annotation)
UMAP 1
UMAP 2
1 Advanced Mesoderm
2 Axial Mesoderm
3 Non-neural Ectoderm
4 Emergent Mesoderm
5 Definitive Endoderm (NP)
6 Epiblast
7 Erythroblasts
8 Yolk-sac Mesoderm
9 Hemogenic Endo Prog
10 Nascent Mesoderm
11 Primitive Streak
12 PGC
13 Amnion
14 Definitive Endoderm (P)
15 Yolk-sac Endoderm
16 Erythro-Myeloid Prog
17 Hemogenic Endothelium
18 Myeloid Prog
b
Human cell type annotation
Myeloid Progenitors
DE(NP)
NNE
PGC
DE(P)
Epiblast
Erythro-Myeloid Progenitors
Erythroblasts
Blood Progenitors
Axial Mesoderm
Amnion
YS Mesoderm
Hypoblast
Emergent Mesoderm
Hemogenic Endothelium
Primitive Streak
Nascent Mesoderm
YS Endoderm
Advanced Mesoderm
Pig cell type annotation
% Allocated
c
Human (Pig annotation)
UMAP 1
UMAP 2
1 Cardiac Mesoderm
2 Caudal Epiblast
3 Mesenchyme 2
4 Mesenchyme 3
5 Intermediate Mesoderm 1
6 Nascent Mesoderm 2
7 Nascent Mesoderm 4
8 Lateral Plate Mesoderm
9 Nascent Mesoderm 1
10 Primitive Streak 2
11 Primitive Streak 3
12 Epiblast 1
13 Epiblast 2
14 Primitive streak 1
15 Epiblast 3
16 Anterior Primitive Streak
17 Node
18 Midgut 2
19 Definitive Endoderm/Foregut
20 Definitive Endoderm/Hindgut
21 Midgut 1
22 ExE Endo 3
23 ExE Endo 1
24 ExE Endo 2
25 InterHypo
26 Posterior Surface Ectoderm
27 ExE Endo 5
28 Hypoblast 2
29 Hypoblast 1
30 ExE Endo 4
31 ExE APOP
32 Anterior Hypoblast/AVE
33 YS Endo
34 ExE Endo 6
35 Epiblast 4
36 Spinal Cord
37 Anterior Surface Ectoderm
38 Amnion
39 Trophoblast
40 Cranial Mesoderm
41 Mesenchyme 1
42 PGC
43 Dermomyotome/Sclerotome
44 Blood Progenitors
45 Haematoendothelial Progenitors
46 Presomitic Mesoderm
47 Anterior Somitic Mesoderm 3
48 Anterior Somitic Mesoderm 2
49 Anterior Somitic Mesoderm 1
50 Intermediate mesoderm 2
51 Pharyngeal Mesoderm
52 Posterior Somitic Mesoderm
53 Allantois
54 ExE Mesoderm 2
55 ExE Mesoderm 1
56 Posterior Mixed mesoderm
57 Nascent Mesoderm 3
d
Mesodermal differentiation
Monkey cell type
ys.Endo2_E26
ys.Endo1_E26
ys.Endo1_E23
VE_E23
Node_E23
ECT_E23
ECT_E20
DE_E23
DE_E20
AM_E23
Epiblast
Axial Mesoderm
DE(P)
DE(NP)
Hypoblast
YS Endoderm
% Allocated
Human cell type
e
Endodermal differentiation
Monkey cell type
ys.Meso2_E26
ys.Meso2_E23
ys.Meso1_E26
ys.Meso1_E23
ys.Meso1_E20
SE2_E23
Pharyg.Meso_E23
PGC_E20
Nas.Meso_E23
Mes_E23
LP.Meso_E23
ExE.Meso_E23
EPI_E20
ECT_E23
ECT_E20
DE_E23
Caud.Meso_E23
AM_E23
Epiblast
Primitive Streak
Nascent Mesoderm
Emergent Mesoderm
Advanced Mesoderm
YS Mesoderm
% Allocated
Human cell type

Supplementary Fig. 6. Homochronicity among monkey and human cell type differentiation. a, UMAPs showing E17-19 human embryo cell types[7] and pig E11.5 to E15 with mouse annotations after reciprocal PCA-based projection onto the human dataset. P, primitive; NP, non-primitive. **b**, Heat map showing the percentage of pig cells of a given cell type allocated as a particular human cell type after label transfer. **c,** As with a with a, pig annotations after reciprocal PCA-based projection onto the human dataset. **d,** Heat maps showing the percentage of human mesodermal cells[7] allocated to a particular monkey cell identity[6] after label transfer. **e,** As with d, except with endodermal cell types. 100% means that all cells of a given cell type were predicted to be analogous to the cell identity in the queried organism.

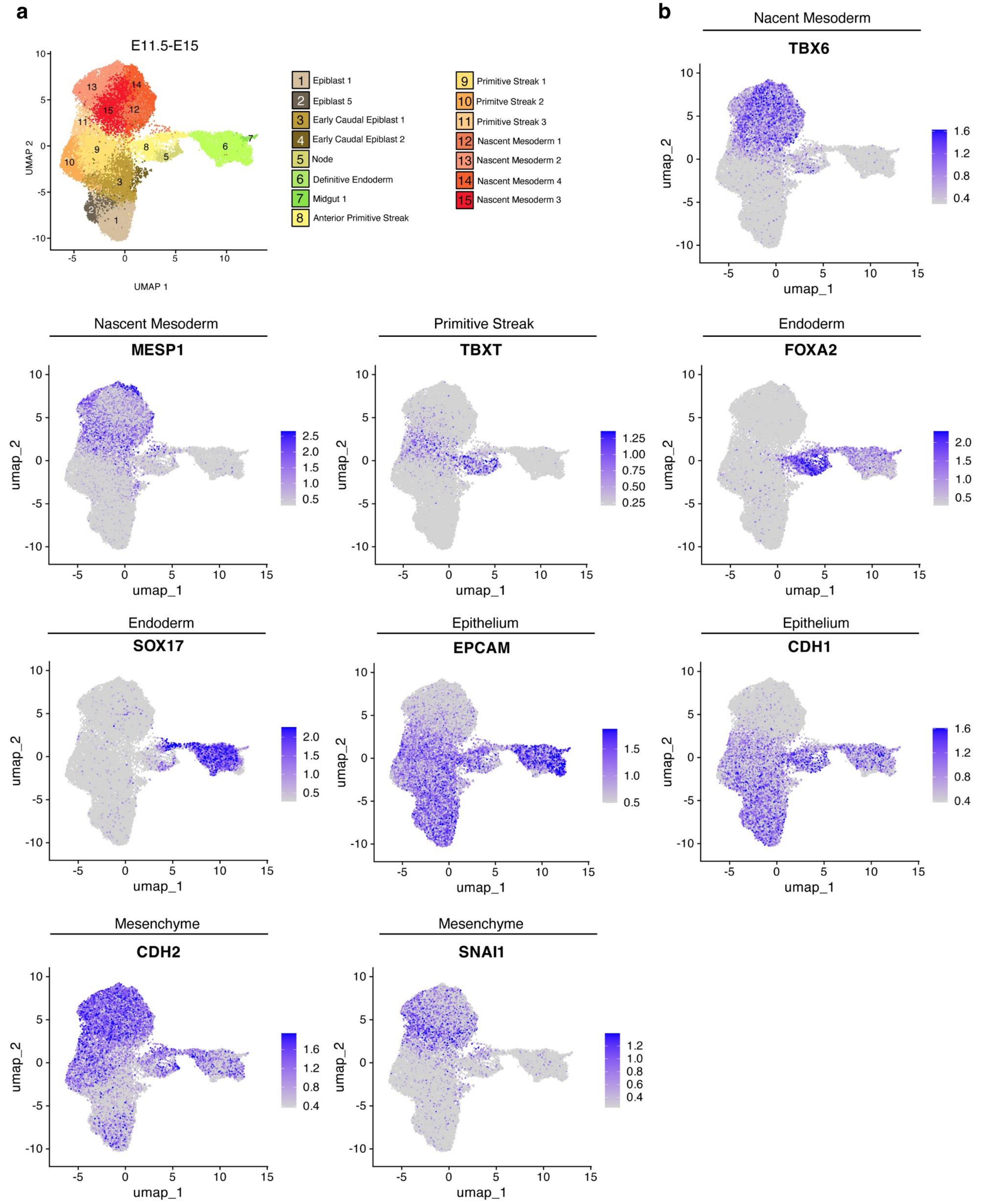

Supplementary Fig. 7. Feature plots of Mesendodermal, Endodermal and EMT-related genes. a, UMAP plot from Fig. 4 showing Epiblast, Primitive Streak, Anterior Primitive Streak, Node, Nascent Mesoderm and Endoderm sub-clusters coloured by cluster. **b**, Feature plots coloured by normalised gene expression of selected marker genes relating to Fig. 4. Feature plot scales were determined by the 5th and 95th percentiles.

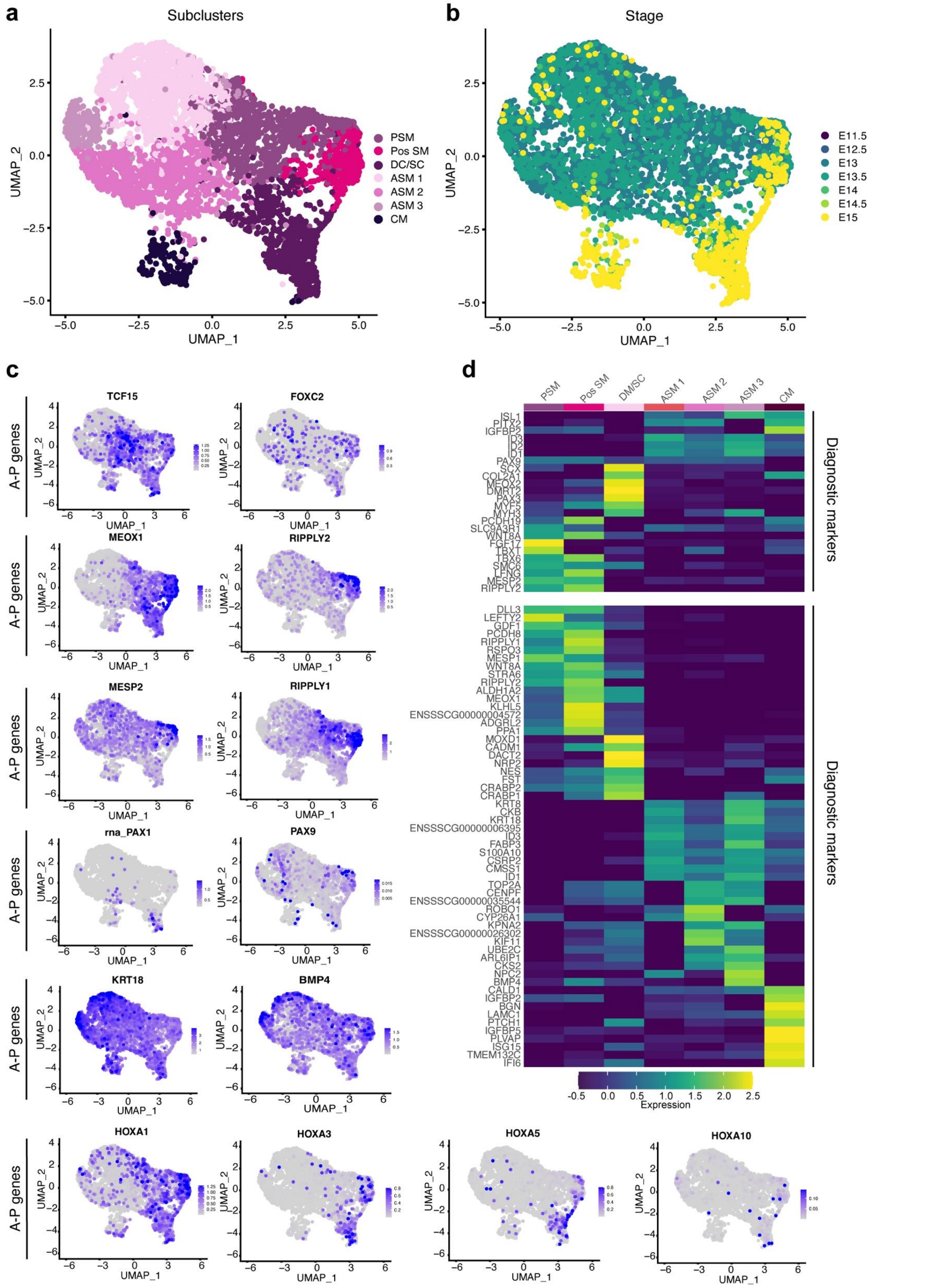

Supplementary Fig. 8. Somitogenesis in porcine embryos. **a**, UMAP plot showing sub-clustering of Presomitic and Somitic mesoderm clusters. 7 subclusters were identified: Presomitic mesoderm (PSM), Posterior somitic mesoderm (Pos SM), Dermomyotome/Sclerotome (DC/SC), Anterior Somitic Mesoderm 1, 2 and 3 (ASM1, ASM2, ASM3), as well as Cranial Mesoderm (CM). **b**, UMAP plot showing cells from a. Cells are coloured by embryonic day. **c**, UMAP feature plots of key markers of somite development. Feature plots coloured by normalised gene expression. Feature plot scales were determined by the 5th and 95th percentiles. **d**, Heat map illustrating the scaled average expression of selected genes. Shown are diagnostic marker genes for each subcluster and the top 10 most significant marker genes.

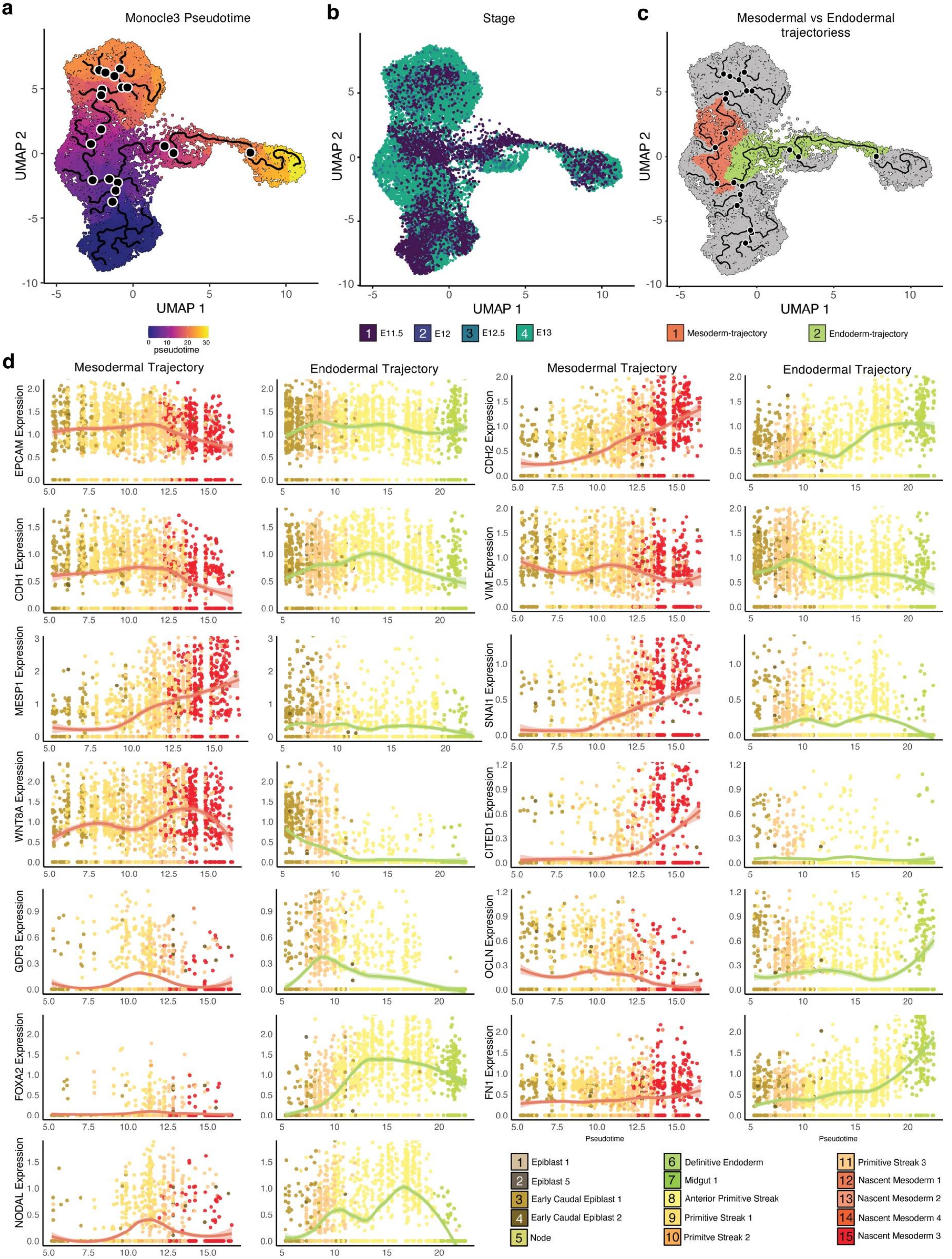

Supplementary Fig. 9. Dynamics of EMT-related genes in mesendoderm and endoderm fated cells. a, UMAP plot showing Epiblast, Primitive Streak, Anterior Primitive Streak, Node, Nascent Mesoderm and Endoderm sub-clusters from Fig.5a with reversed graph embedding trajectories projected on top using Monocle3, coloured by Monocle3 pseudo time. Black nodes mark trajectory branching points. **b**, UMAP coloured by developmental time point. **c**, UMAP with reversed graph embedding trajectories projected on top, cells associated with nodes going in the nascent mesoderm trajectory are coloured in red, while those associated with the endoderm trajectory are coloured in green. **d,** Scatter plots showing gene expression and Monocle3 pseudo time values in mesodermal (left) vs endodermal (right) fated cells.

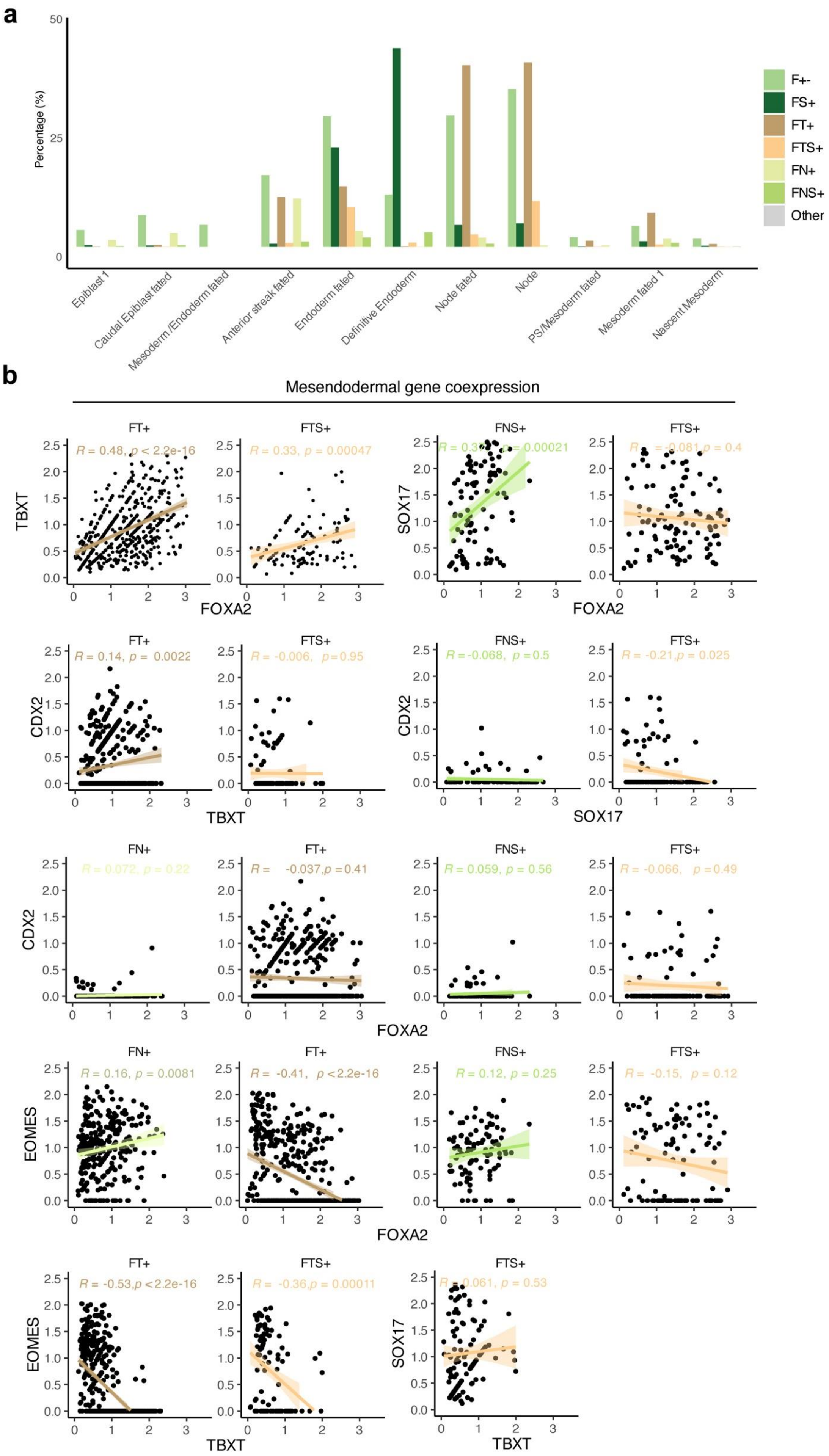

Supplementary Fig. 10. Mesodermal and endodermal genes are mutually exclusive. a, Bar graphs illustrating cells categorised by FOXA2, NANOG, TBXT and SOX17 co-expression status and their relative percentage abundance in E11.5-13 epiblast, caudal epiblast, PS, APS, node and DE fated cells. **b**, Scatter plots showing mesoderm and endoderm associated transcription factor co-expression of F/N/T/S categorised cells from Fig 4.a & Extended Data Fig.8a. Correlation coefficient (R) and adjusted p-value (p) following Pearson correlation test indicated.

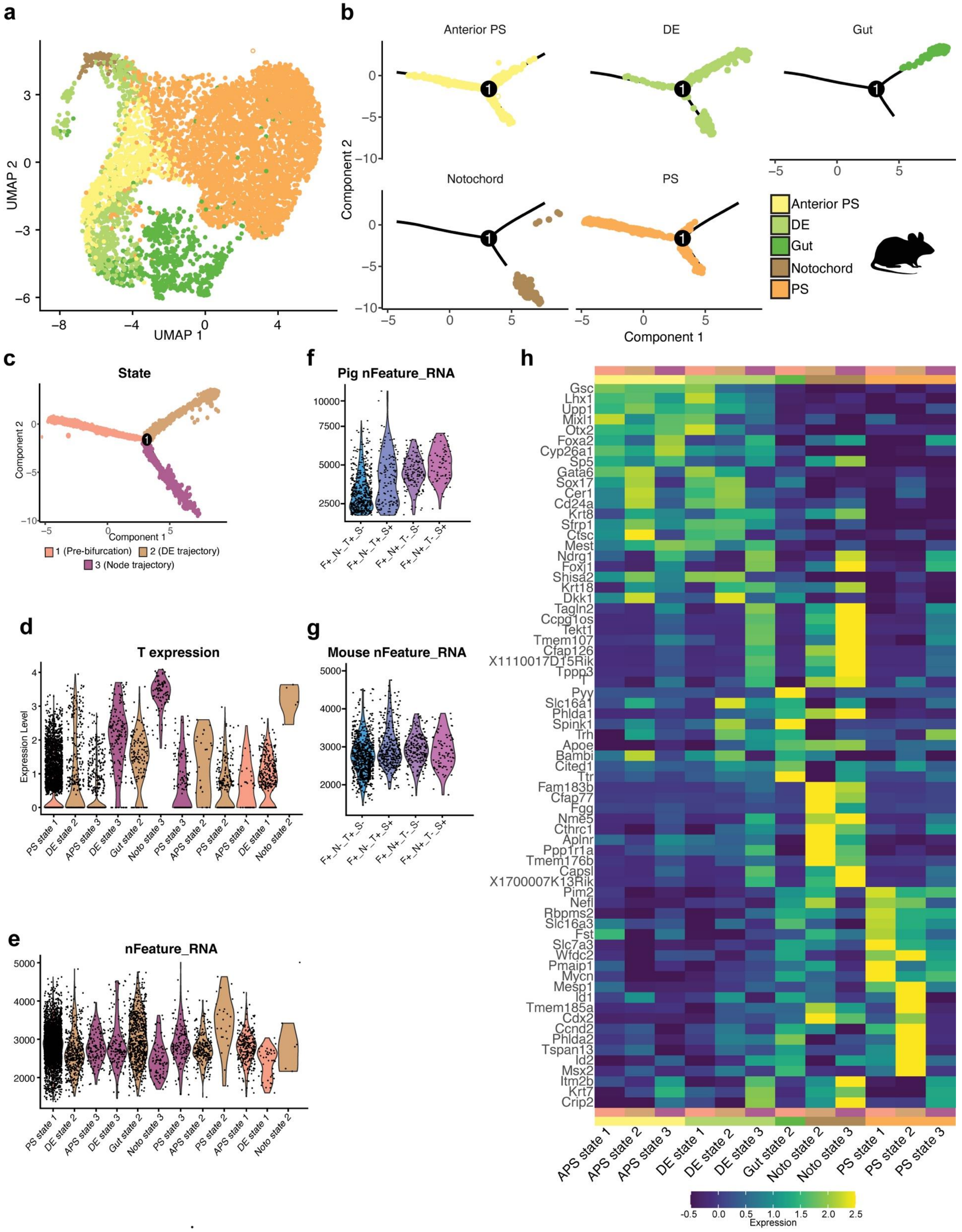

Supplementary Fig. 11. Endoderm forms from TBXT-low cells in mice. **a**, UMAP plot showing PS, APS, Notochord, DE and Gut clusters (5,860 cells) from Pijuan-Sala et al[2], coloured by cell type. **b**, Force-directed graph of the cells illustrated in a, after pseudo-temporal ordering using Monocle2. Force-directed graphs are split by cell type. **c** Force-directed graph of the cells illustrated in a and b coloured by state. State 1 represents cells that have not committed to notochord or DE/Gut fates. State 2 represents cells moving toward a DE/gut fate. State 3 represents cells moving toward a Notochord fate. **d**, Violin plots of T expression in the cell types defined in c. **e**, Violin plots showing the number of mapped genes in each cell type defined in d. **f**, Violin plots showing the number of mapped genes in the cell types of interest defined in Figs 5-7. **g**, Violin plots showing the number of mapped genes in the mouse-equivalent cell types shown in f. Both mouse and pig DE/Node progenitors show a comparable number of mapped genes. **h**, Heat map illustrating the scaled mean expression of the top 10 most significant DEGs between the cell types from c-e.

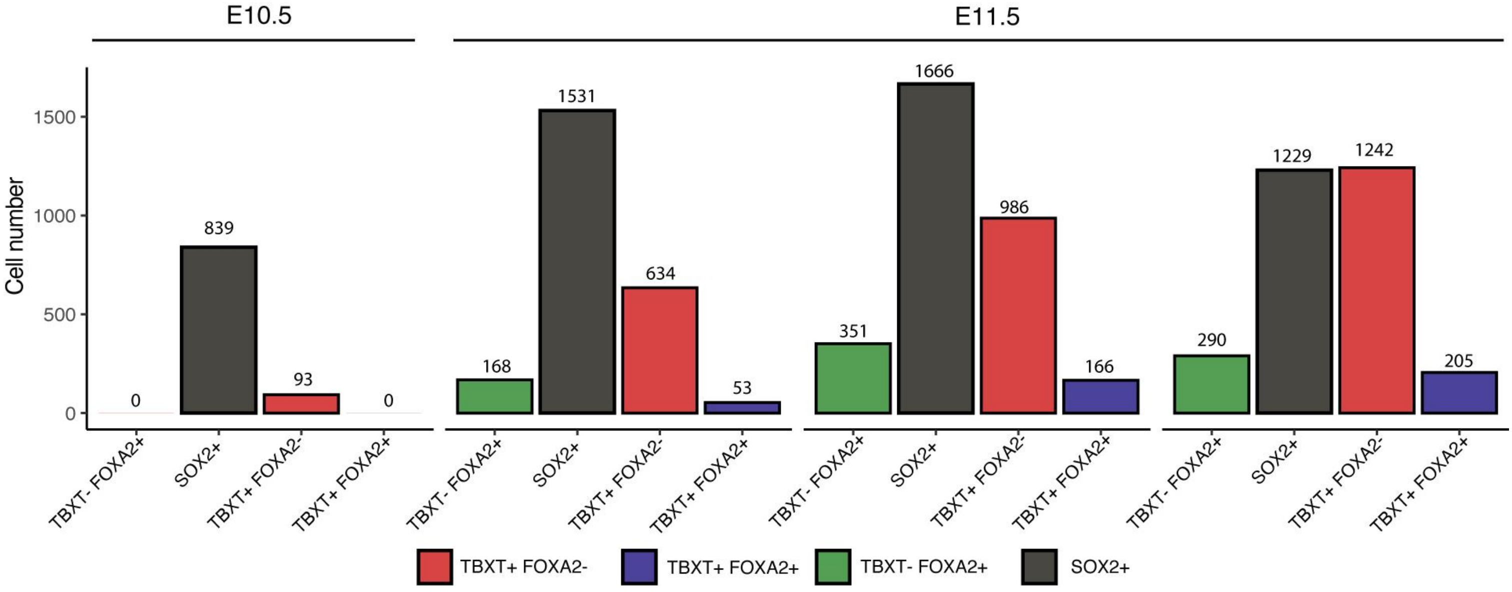

Supplementary Fig. 12. Quantification of 3D segmentation of whole mount porcine embryo immuno-staining from figure 5c. Segmentation via Trackmate and Stardist. Thresholding was achieved via Otsu's method within Fiji.

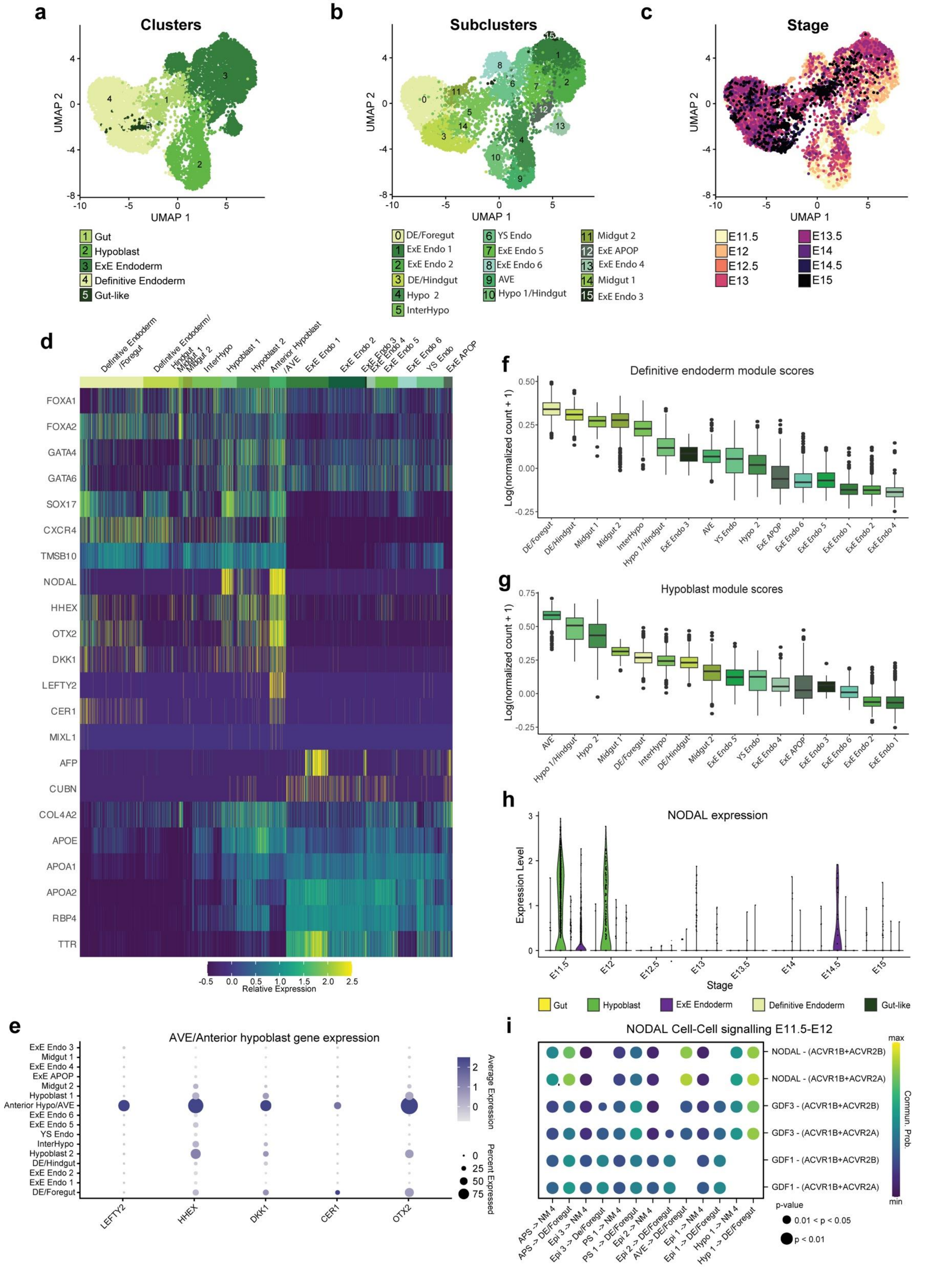

a
Clusters
UMAP 2
UMAP 1
1 Gut
2 Hypoblast
3 ExE Endoderm
4 Definitive Endoderm
5 Gut-like
b
Subclusters
0 DE/Foregut
1 ExE Endo 1
2 ExE Endo 2
3 DE/Hindgut
4 Hypo 2
5 InterHypo
6 YS Endo
7 ExE Endo 5
8 ExE Endo 6
9 AVE
10 Hypo 1/Hindgut
11 Midgut 2
12 ExE APOP
13 ExE Endo 4
14 Midgut 1
15 ExE Endo 3
c
Stage
E11.5
E12
E12.5
E13
E13.5
E14
E14.5
E15
d
Definitive Endoderm /Foregut
Definitive Endoderm/ Hindgut
Midgut 1
Midgut 2
InterHypo
Hypoblast 1
Hypoblast 2
Anterior Hypoblast /AVE
ExE Endo 1
ExE Endo 2
ExE Endo 3
ExE Endo 4
ExE Endo 5
ExE Endo 6
YS Endo
ExE APOP
FOXA1
FOXA2
GATA4
GATA6
SOX17
CXCR4
TMSB10
NODAL
HHEX
OTX2
DKK1
LEFTY2
CER1
MIXL1
AFP
CUBN
COL4A2
APOE
APOA1
APOA2
RBP4
TTR
Relative Expression
e
AVE/Anterior hypoblast gene expression
Average Expression
Percent Expressed
LEFTY2
HHEX
DKK1
CER1
OTX2
f
Definitive endoderm module scores
Log(normalized count + 1)
g
Hypoblast module scores
h
NODAL expression
Expression Level
Stage
Gut
Hypoblast
ExE Endoderm
Definitive Endoderm
Gut-like
i
NODAL Cell-Cell signalling E11.5-E12
NODAL - (ACVR1B+ACVR2B)
NODAL - (ACVR1B+ACVR2A)
GDF3 - (ACVR1B+ACVR2B)
GDF3 - (ACVR1B+ACVR2A)
GDF1 - (ACVR1B+ACVR2B)
GDF1 - (ACVR1B+ACVR2A)
Commun. Prob.
max
min
p-value
0.01 < p < 0.05
p < 0.01

Supplementary Fig. 13. The hypoblast is a major source of signaling and contributes to DE. **a**, UMAP plot showing DE, gut-like, gut, hypoblast and ExE Endoderm subclusters coloured by cell-type. **b**, UMAP plot showing embryonic and extraembryonic endodermal sub-clusters coloured by cluster. **c**, UMAP plot showing cells from a&b coloured by timepoint, showing stage specificity of clusters. **d**, Heat map illustrating the cluster-scaled mean expression of the top 10 most significant newly discovered marker genes for each subcluster. **e,** Dot plot showing the average expression and percentage of cells expressing AVE/Anterior hypoblast related genes. **f&g**, Box and whisker plots showing the respective DE and hypoblast module scores in each subcluster. Modules were defined as the top 100 most significant markers of the E11.5 DE or hypoblast (See Methods). **h**, Violin plot showing the expression of *NODAL* at each time point in the clusters from a. **i**, Predicted *NODAL* cell-cell signalling between selected cell types in E11.5-E12 embryos.

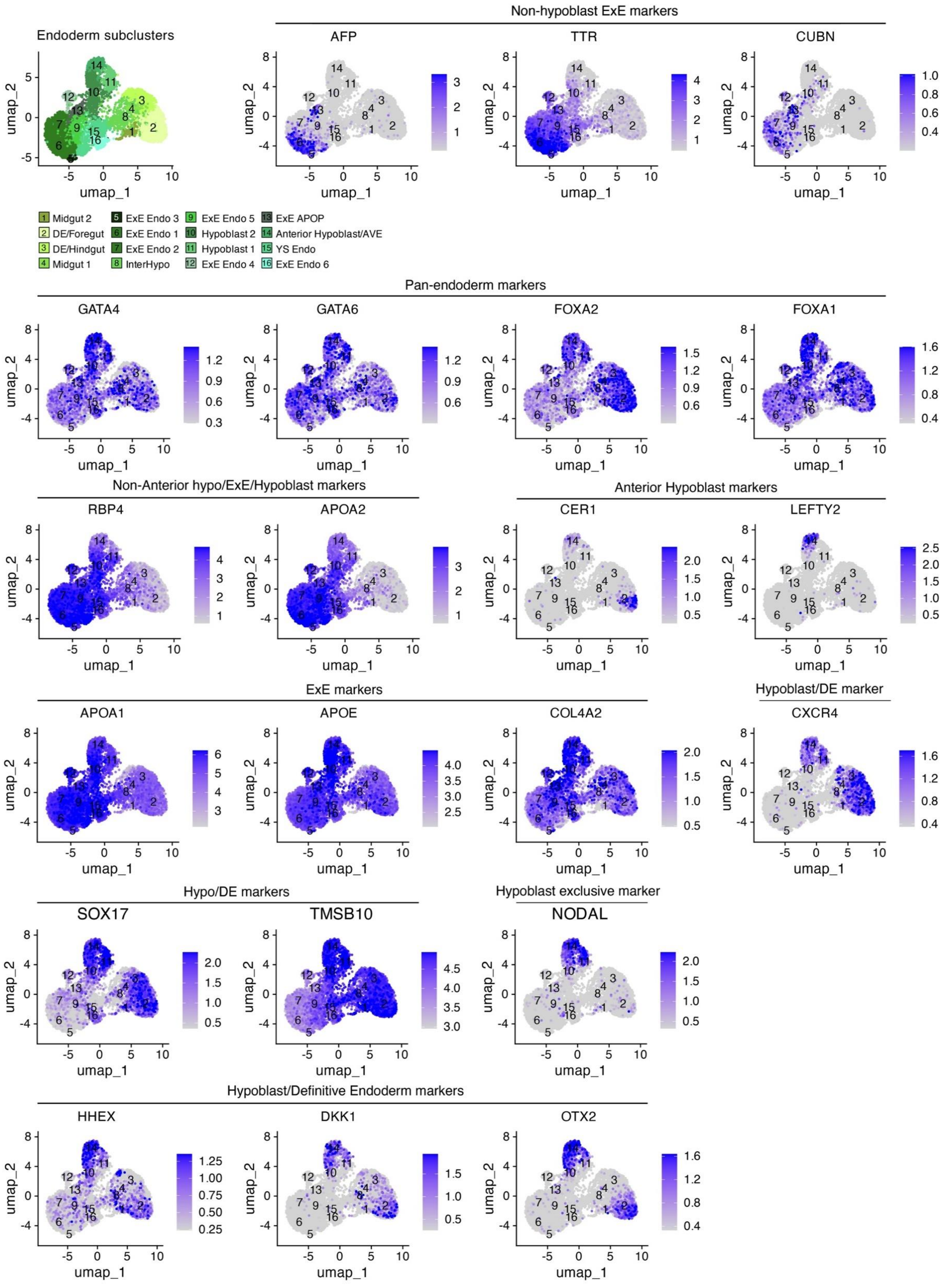

Supplementary Fig. 14. Feature plots to verify Endodermal subtypes. UMAP plot (top left) showing embryonic and extraembryonic endoderm sub-clusters coloured by cluster and plots coloured by normalised gene expression of selected marker genes relating to Supplementary Figure 15. Feature plot scales were determined by the 5th and 95th percentiles.

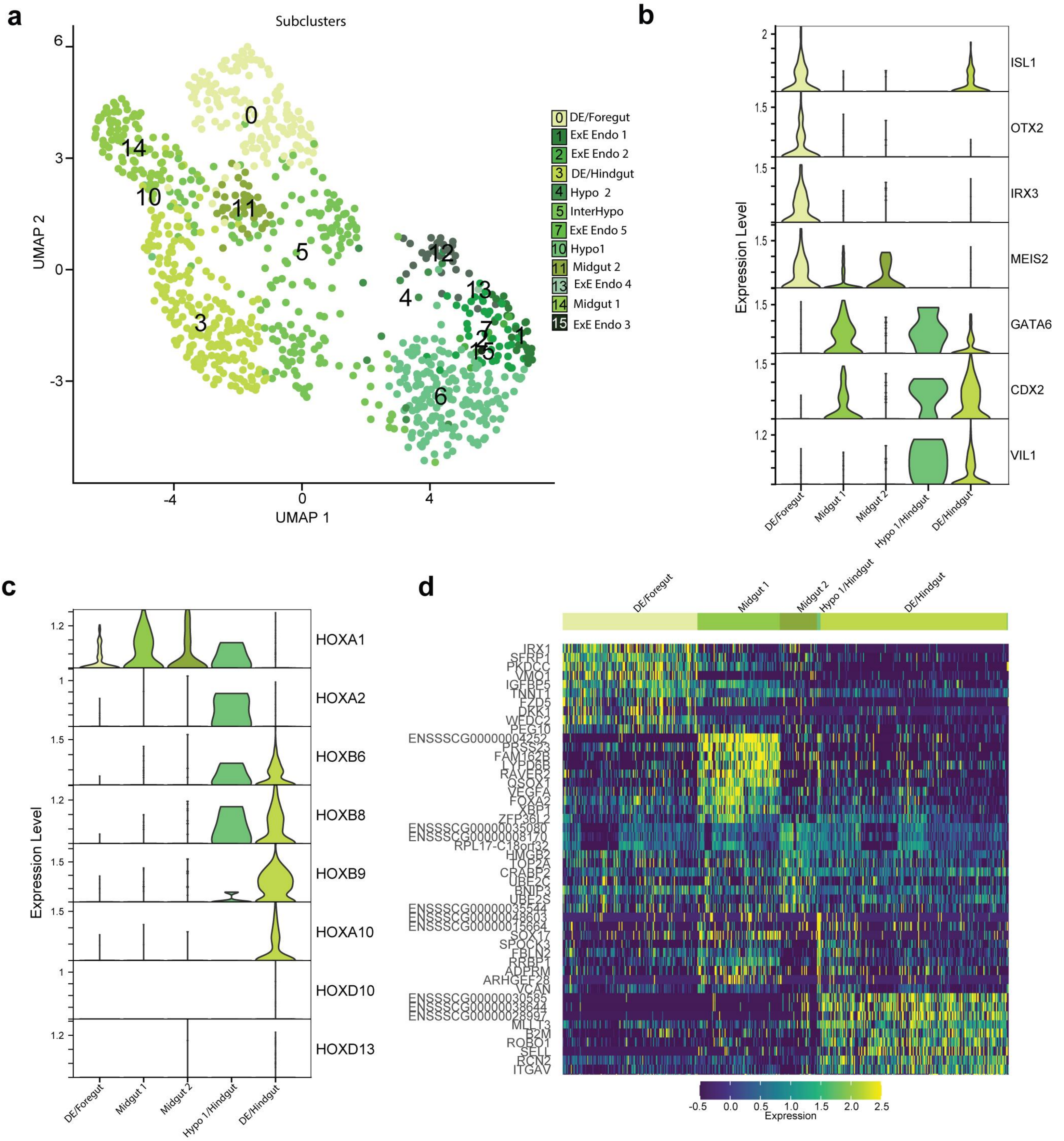

Supplementary Fig. 15. Mature gut types emerge between E14-E15. **a**, UMAP plot showing embryonic and extraembryonic sub-clusters from Fig 10 from E14-E15. **b**, Violin plots of gene expression showing late endodermal subclusters show specific expression of gut markers described by Nowotschin et al[42]. **c**, Violin plots of Hox gene expression showing the distinct anterior-posterior positioning of gut subclusters. **d**, Heat map illustrating the scaled mean expression of the top 10 most significant markers of each gut subcluster.

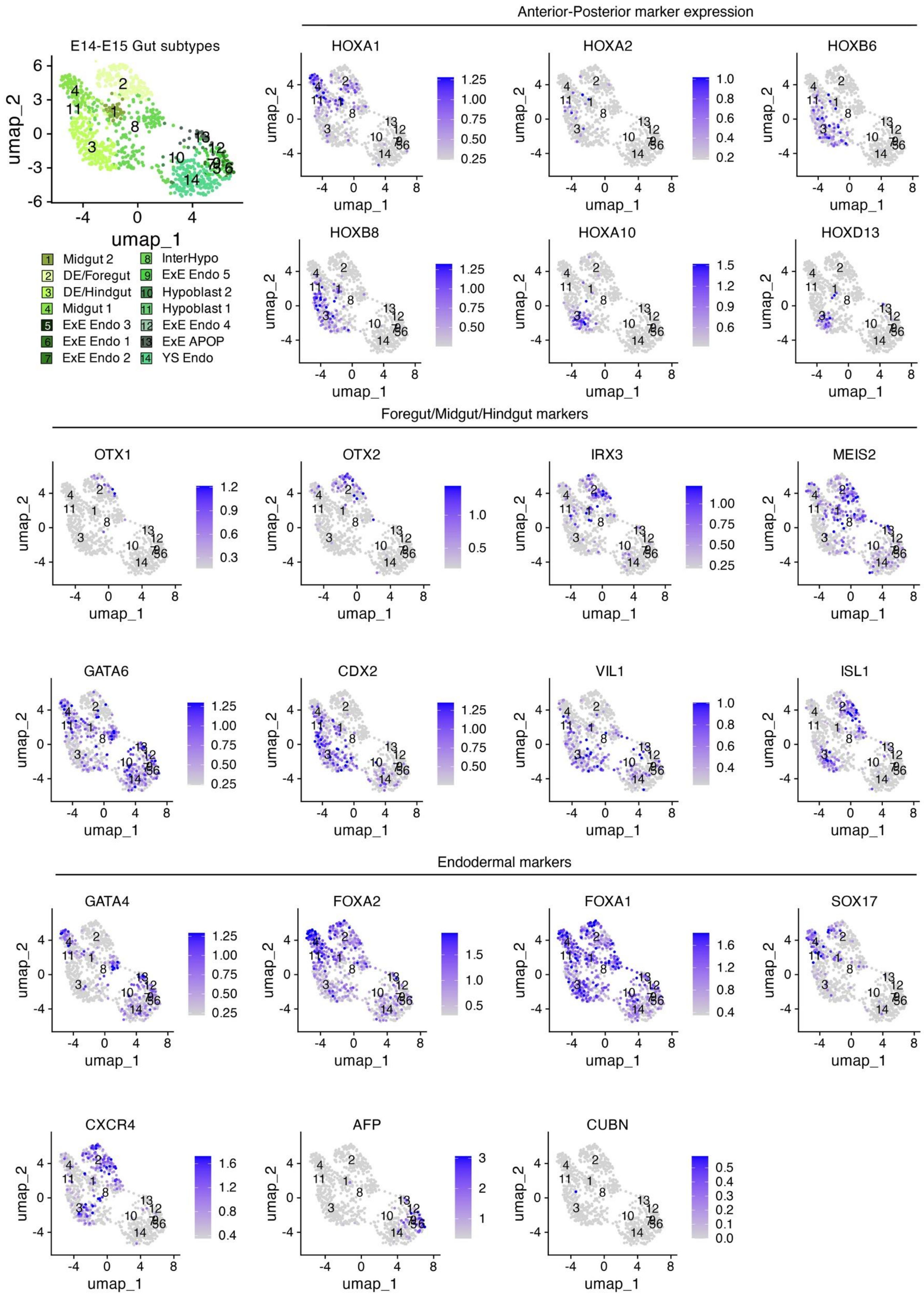

Supplementary Fig. 16. Feature plots to verify Gut subtypes. UMAP plot showing E14-E15 gut sub-types identified in Extended Data Fig. 11 and UMAP plots coloured by normalised gene expression of selected marker genes.

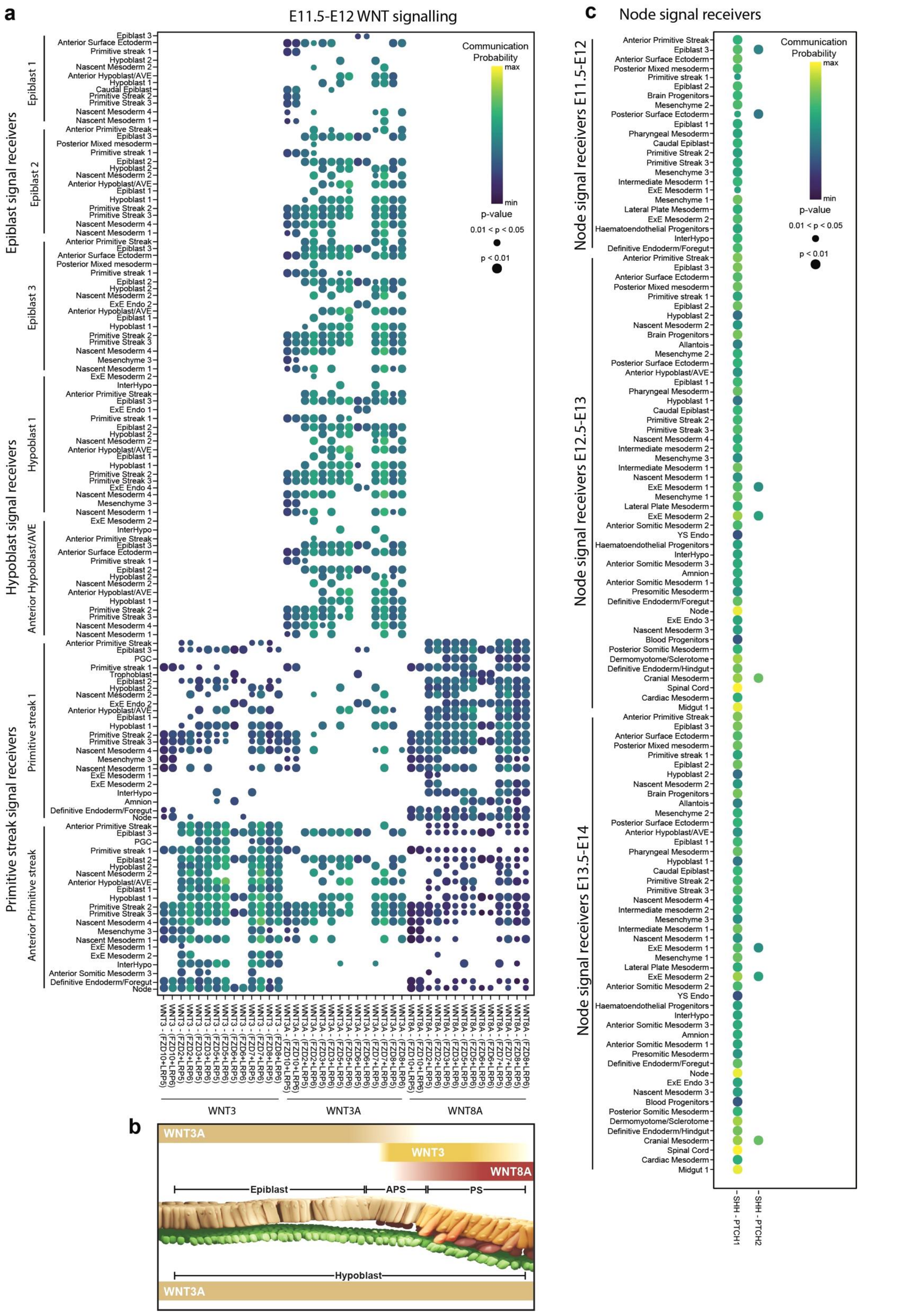

Supplementary Fig. 17. WNT and Nodal signaling in early pig gastrula embryos. **a**, Predicted canonical WNT3, WNT3A and WNT8A cell-cell signalling in E11.5-12 Epiblast, Hypoblast and PS clusters. **b**, Schematic summarising a. **c**, Predicted Hedgehog cell-cell signalling between selected cell types in E11.5-14 embryos.

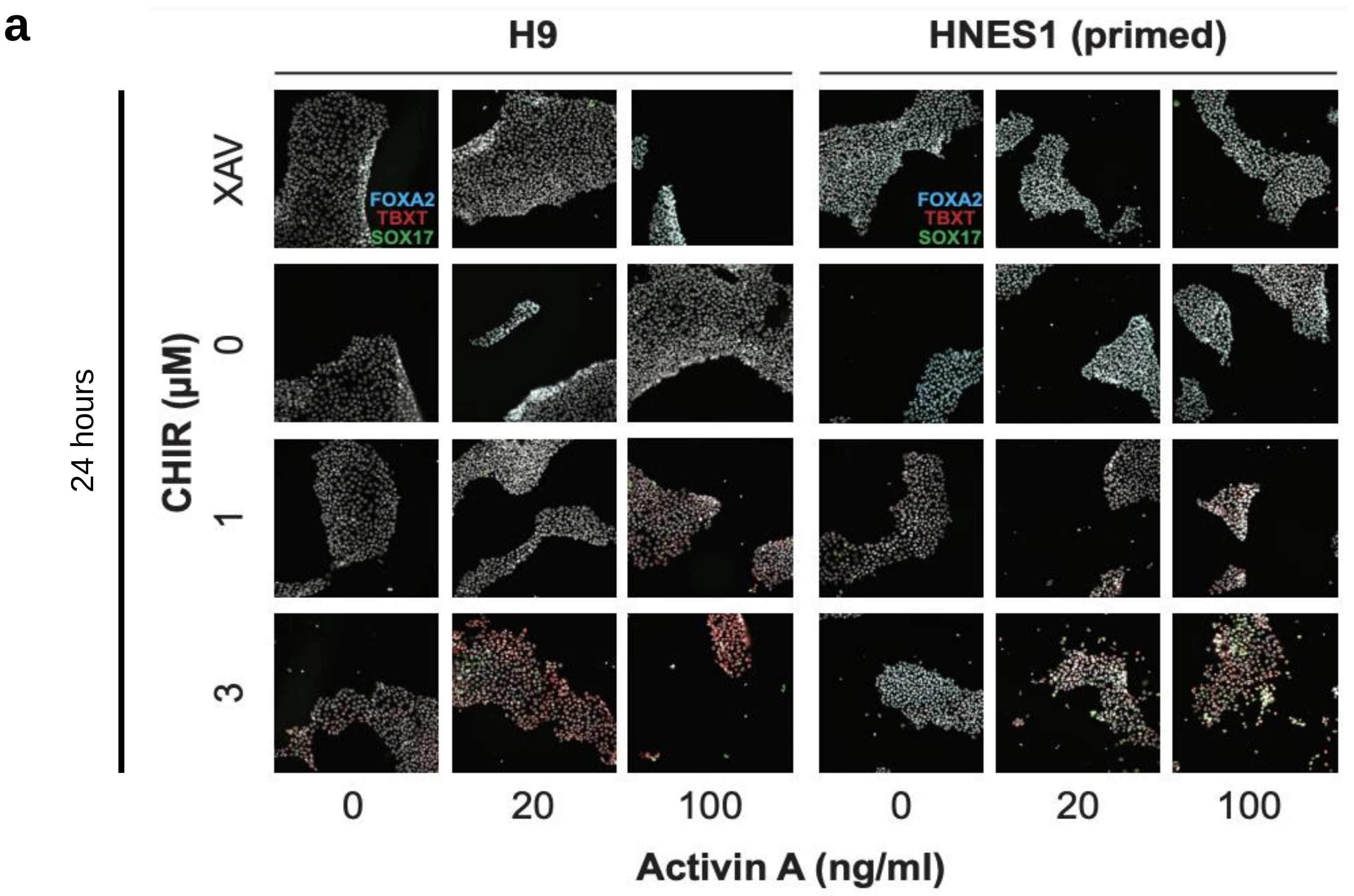

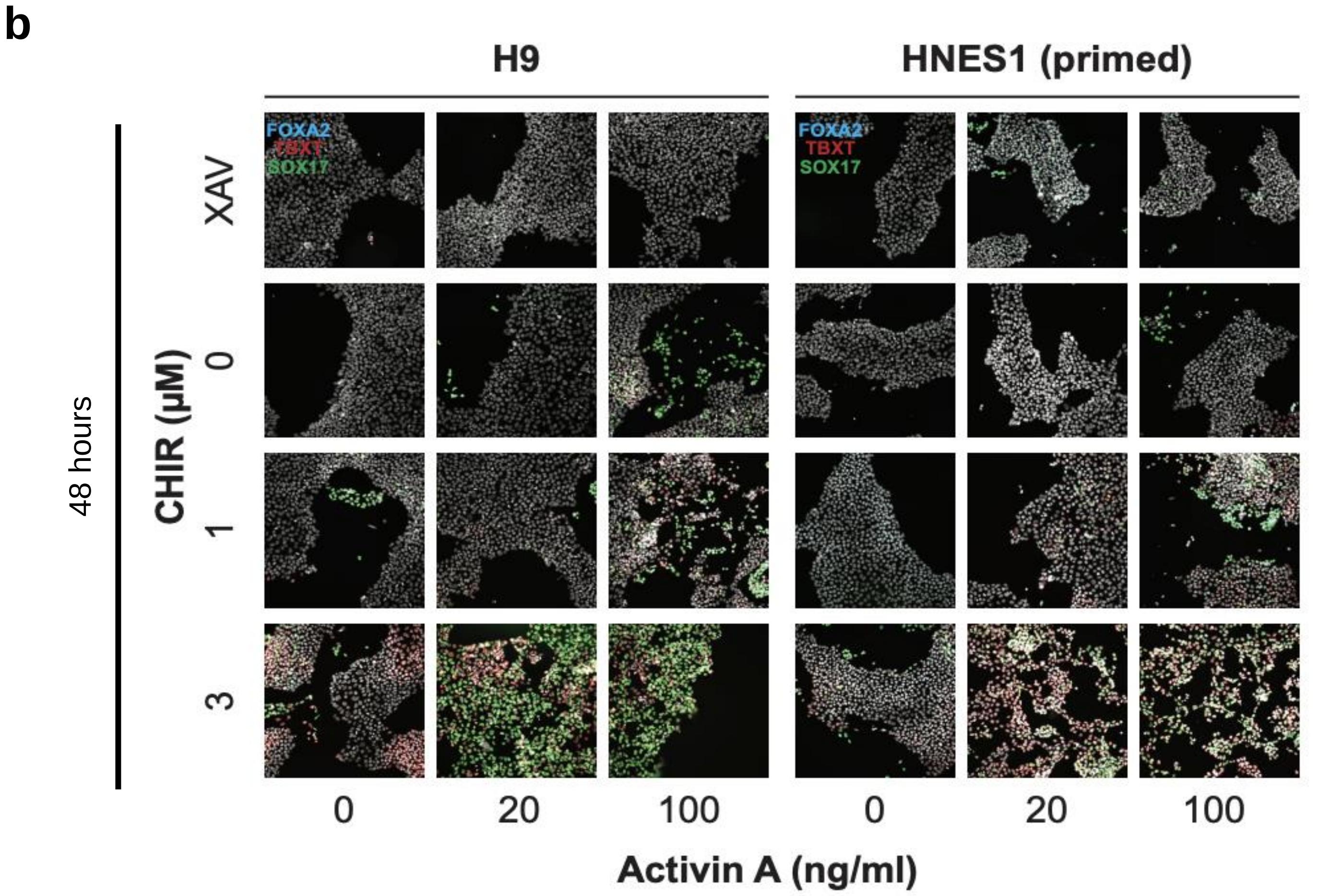

Supplementary Fig. 18. Co-expression of mesodermal and endoderm markers. Representative images of H9 and HNES1 hESC lines after treatments with differing amounts of XAV, CHIR and/or Activin A. **a,** 24hrs after treatment **b,** 48hours after treatment. FOXA2 (blue), TBXT (red) and SOX17 (green) expression.

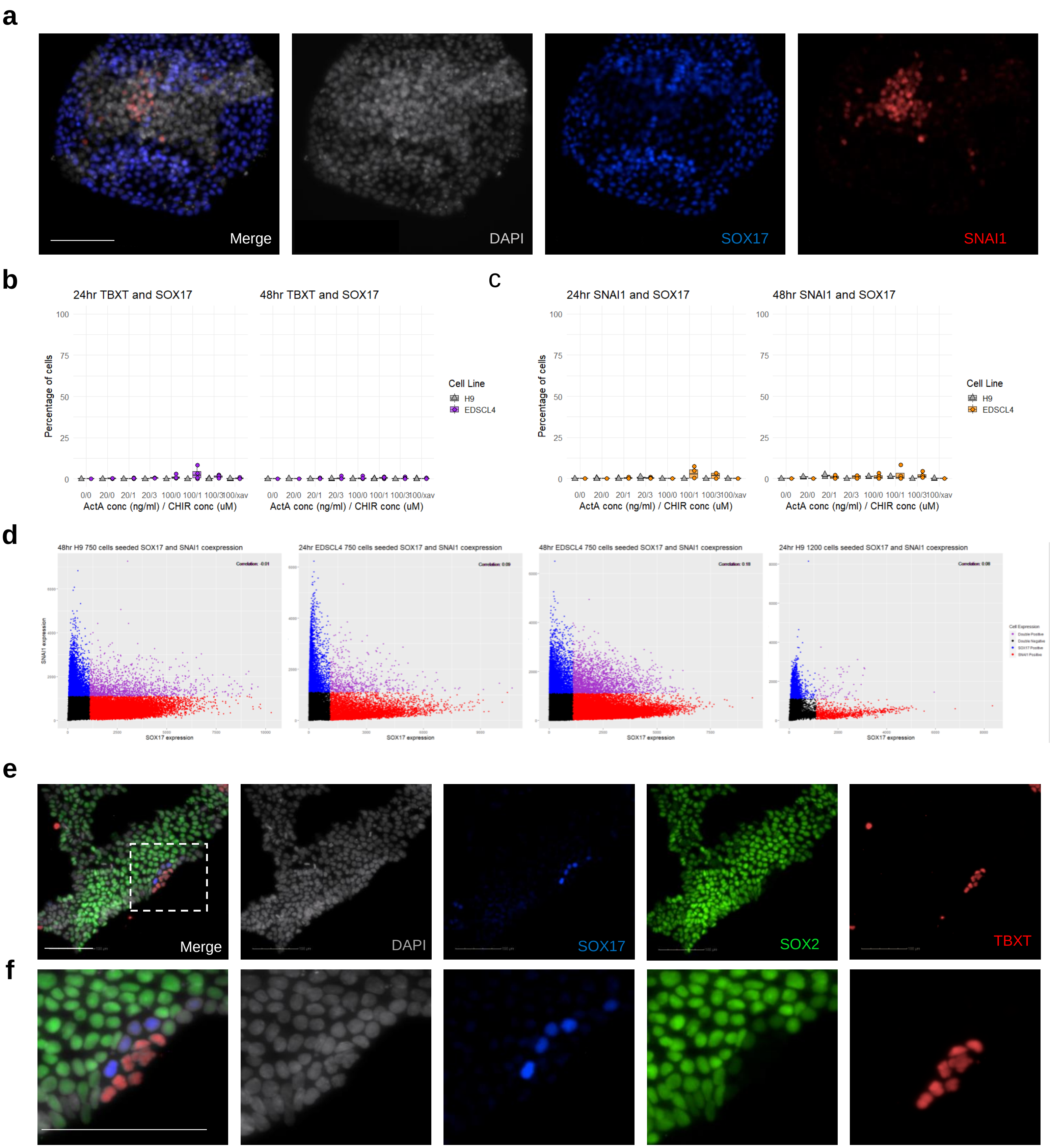

Supplementary Fig. 19. Co-expression of endoderm markers. a, Representative images of EDSCL4 after 48 hours of induction with ActA (100ng/ml) and CHIR (1µM). Scale bar: 100µm. **b** Quantification of SOX17 and TBXT co-expression across the 2D experiments. **c**, Quantification of SOX17 and SNAI1 co-expression across the 2D work. **d,** Selection of correlation graphs between SNAI1 and SOX17. Correlation value calculated via Pearson's correlation. **e,** representative images of EDSCL4 after 8 hours of induction with ActA (100ng/ml) and CHIR (1µM). Scale bar: 100µm. **f** enlargement of ROI (dotted box in e). Scale bar: 100µm.

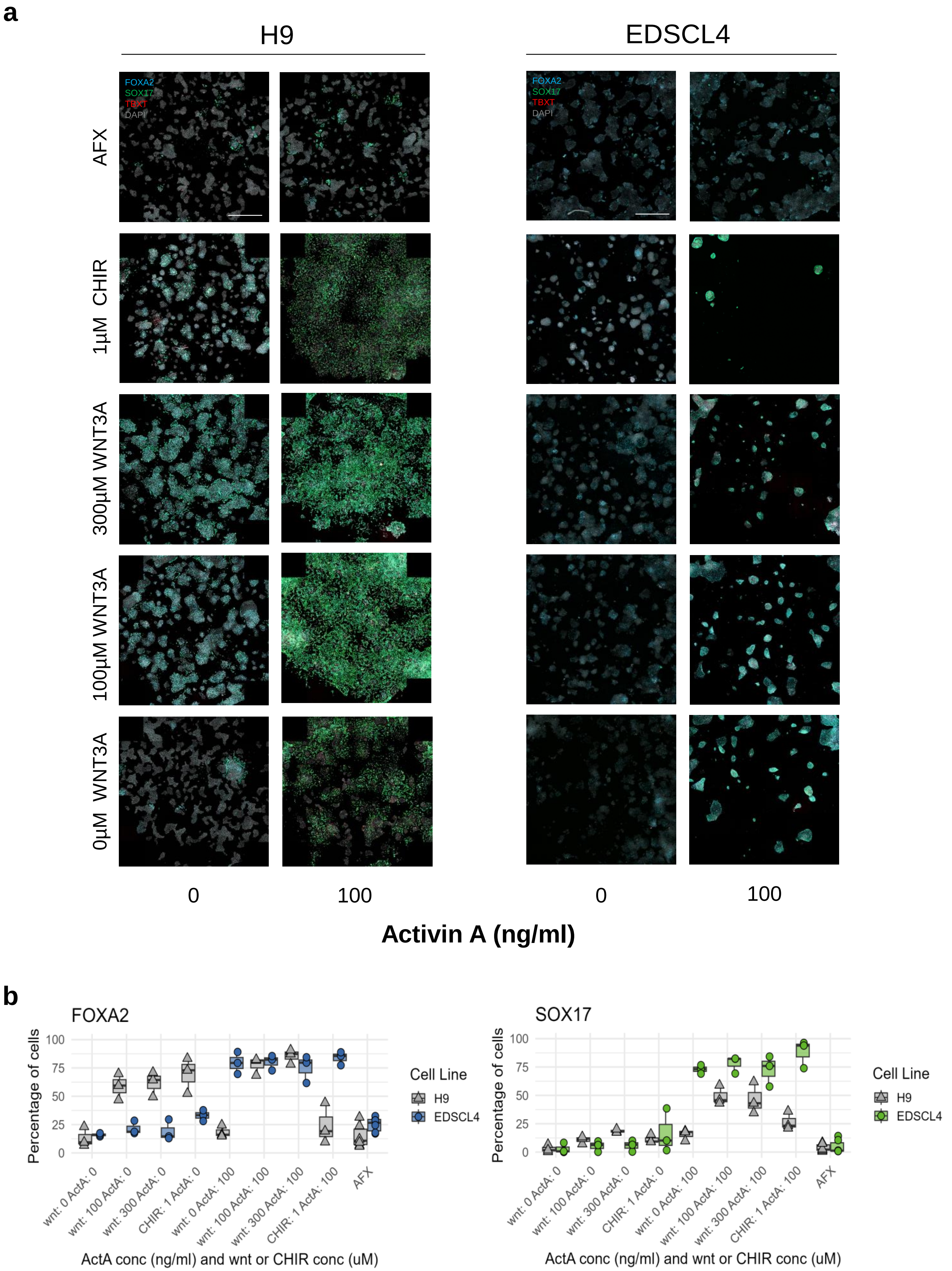

Supplementary Fig. 20. Effect of Activin A and WNT on endoderm formation in 2D differentiation. **a,** Overview of each well within human (H9) and porcine (EDSCL4) 2D WNT3A and Activin A differentiation experiments. Green: SOX17, blue : FOXA2, grey : DAPI, red : TBXT. Scale bar: 1000um. **b,** Quantification of SOX17 and FOXA2 after 48 hrs incubation with WNT3A, CHiR and ActA (n=3).

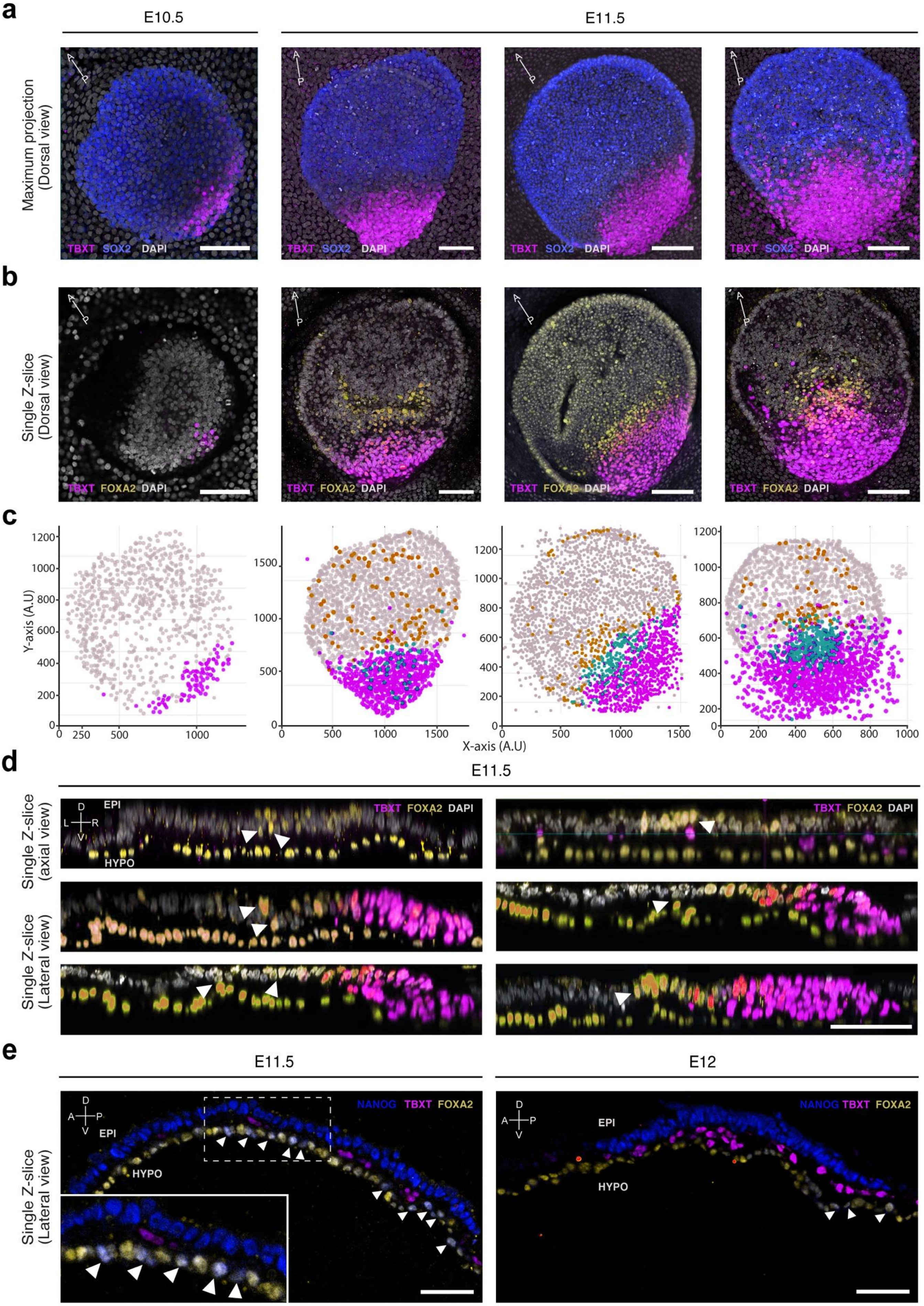

a
E10.5
E11.5
Maximum projection (Dorsal view)
TBXT SOX2 DAPI
b
Single Z-slice (Dorsal view)
TBXT FOXA2 DAPI
c
Y-axis (A.U)
X-axis (A.U)
d
E11.5
Single Z-slice (axial view)
Single Z-slice (Lateral view)
EPI
HYPO
TBXT FOXA2 DAPI
e
E11.5
E12
Single Z-slice (Lateral view)
NANOG TBXT FOXA2
EPI
HYPO

Supplementary Fig. 21. FOXA2 and TBXT domains are spatially separated (Colour-blind friendly). **a**, Maximum intensity projection (Dorsal view) of E10.5 to E11.5 porcine embryos showing TBXT and SOX2 expression. E11.5 embryos are ordered left to right by age. E, Embryonic day. **b**, Single z-slice of the embryos shown in a, showing FOXA2 and TBXT expression. Scale bar: 50µm. **c**, *In Silico* representations of embryos following 3D segmentation of embryos from **a** and **b**. **d**, Axial and lateral reconstructed sections of embryos stained for FOXA2 and TBXT. Epiblast layer is oriented above the hypoblast/DE layer. White arrowheads indicate FOXA2+ TBXT- cells that are spatially separated from the TBXT domain. Scale bar: 50µm. **e**, Lateral sections of E11.5 and E12 embryos, showing expression of NANOG, FOXA2 and TBXT. Epiblast layer is oriented above the hypoblast/DE layer. White arrowheads indicate NANOG+ cells. Scale bar: 50µm.

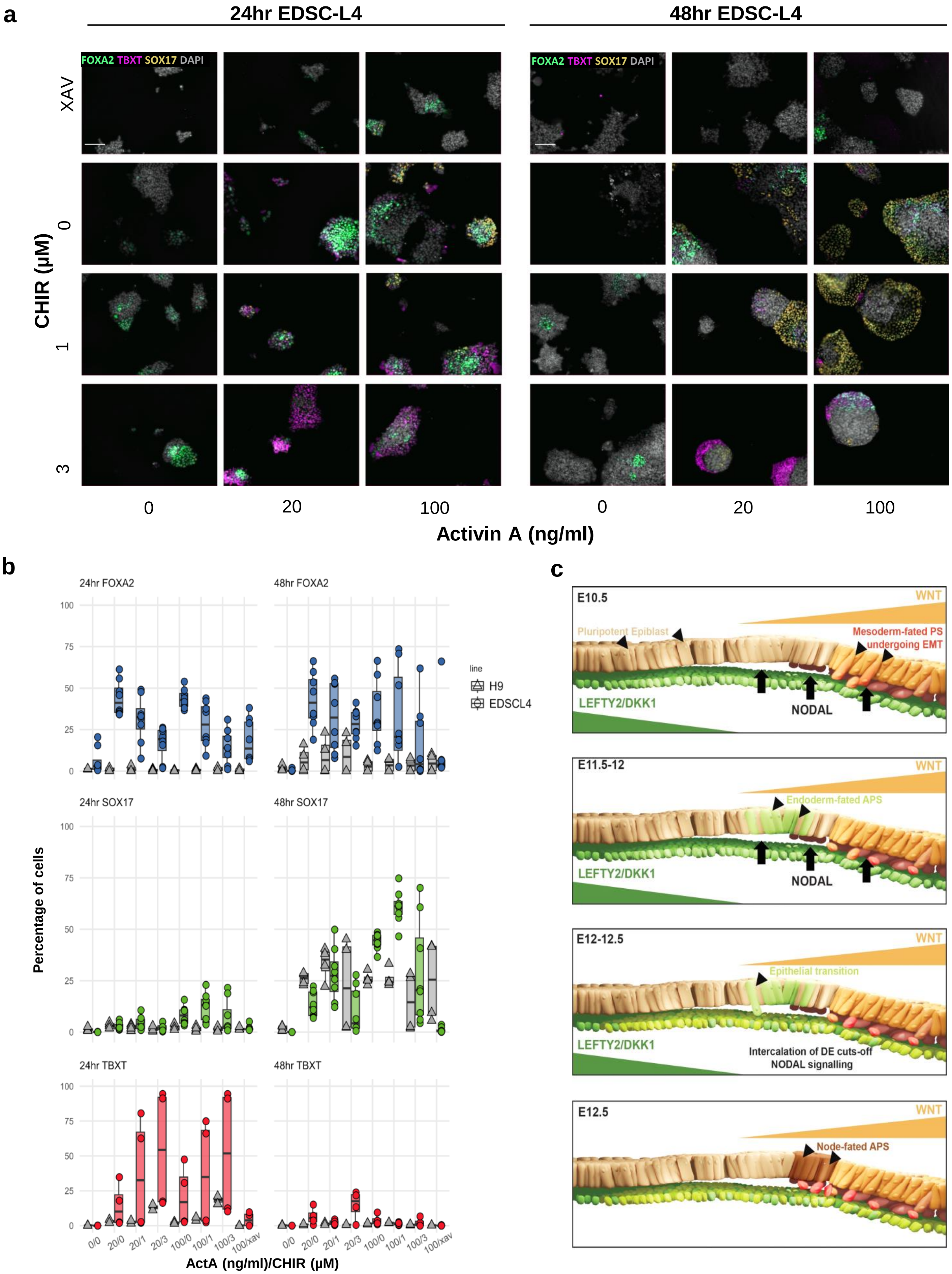

Supplementary Fig. 22. Effect of Activin A and WNT signaling in pig EpiSC and hESC (Colour-blind friendly). **a,** Representative images depicting differentiation conditions for EDSCL4. Images were captured using an Operetta CLS high-throughput microplate imager. Scale bar: 200 µm. **b,** Box plots summarizing 2D differentiation experiments. Data is normalised to well background signal and results represent biological triplicates. Centre line represents median, minima and maxima hinges represent the 25th and 75th percentiles respectively. **c,** Proposed model of epiblast-DE differentiation in pig embryos.
