# Supplementary material for: A single-cell atlas of pig gastrulation as a resource for comparative embryology
Source: Nat Commun. 2024 Jun 18;15:5210. doi: 10.1038/s41467-024-49407-6 (PMC11189408; doi:10.1038/s41467-024-49407-6)
Supplement: Supplementary file 3 — Description of Additional Supplementary Files [file 41467_2024_49407_MOESM3_ESM.pdf]

Description of Additional Supplementary Files

File Name: Supplementary Data 1

Description: A referenced list of markers used for cell type identification.

File Name: Supplementary Data 2

Description: Top markers of cell types identified in this paper. The differential expression analysis presented was performed using the FindAllMarkers function from the Seurat package which uses a two-sided Wilcoxon Rank Sum test. P-values were adjusted for multiple testing using the Bonferroni correction method.

File Name: Supplementary Data 3

Description: Top markers of cell subtypes identified in this paper. The differential expression analysis presented was performed using the FindAllMarkers function from the Seurat package which uses a two-sided Wilcoxon Rank Sum test. P-values were adjusted for multiple testing using the Bonferroni correction method.

File Name: Supplementary Data 4

Description: Conserved and divergent markers of cell types between pig and monkey embryos. The analysis presented was performed using the FindConservedMarkers function from the Seurat package which uses a two-sided Wilcoxon Rank Sum test. P-values were adjusted for multiple testing using the Bonferroni correction method.

File Name: Supplementary Data 5

Description: Conserved and divergent markers of cell types between pig and mouse embryos. The analysis presented was performed using the FindConservedMarkers function from the Seurat package which uses a two-sided Wilcoxon Rank Sum test. P-values were adjusted for multiple testing using the Bonferroni correction method.

File Name: Supplementary Data 6

Description: Conserved and divergent markers of cell types between mouse and monkey embryos. The analysis presented was performed using the FindConservedMarkers function from the Seurat package which uses a two-sided Wilcoxon Rank Sum test. P-values were adjusted for multiple testing using the Bonferroni correction method.

File Name: Supplementary Data 7

Description: Differentially expressed genes in FOXA2, NANOG, TBXT and SOX17 expressing pig embryonic cells. The differential expression analysis presented was performed using the FindAllMarkers function from the Seurat package which uses a two-sided Wilcoxon Rank Sum test. P-values were adjusted for multiple testing using the Bonferroni correction method.

File Name: Supplementary Data 8

Description: Differentially expressed genes in FOXA2, NANOG, TBXT and SOX17 expressing mouse embryonic cells. The differential expression analysis presented was performed using the FindAllMarkers function from the Seurat package which uses a two-sided Wilcoxon Rank Sum test. P-values were adjusted for multiple testing using the Bonferroni correction method.

File Name: Supplementary Data 9

Description: Differentially expressed genes in endodermal subtypes across all time points in pig embryos. The differential expression analysis presented was performed using the FindAllMarkers function from the Seurat package which uses a two-sided Wilcoxon Rank Sum test. P-values were adjusted for multiple testing using the Bonferroni correction method.

File Name: Supplementary Data 10

Description: Differentially expressed genes in mature endodermal subtypes across E14-E15 in pig embryos. The differential expression analysis presented was performed using the FindAllMarkers function from the Seurat package which uses a two-sided Wilcoxon Rank Sum test. P-values were adjusted for multiple testing using the Bonferroni correction method.

File Name: Supplementary Movie 1

Description: A lateral fly-through of an E11.5 pig embryo stained for FOXA2, TBXT and DAPI (Embryo 1).

File Name: Supplementary Movie 2

Description: A lateral fly-through of an E11.5 pig embryo stained for FOXA2, TBXT and DAPI (Embryo 2).

File Name: Supplementary Movie 3

Description: A lateral fly-through of an E11.5 pig embryo stained for FOXA2, TBXT and DAPI (Embryo 3).
